# Development and Characterization of a Plant-Based Chicken Nugget Analogue Based on Extruded Sacha Inchi Cake, Textured Soy Protein, and Wheat Gluten

**DOI:** 10.3390/molecules31101601

**Published:** 2026-05-10

**Authors:** Jersy J. Asto-Mercado, Carlos Elías-Peñafiel, Bettit Salvá-Ruíz, Christian R. Encina-Zelada

**Affiliations:** 1 Departamento de Tecnología de Alimentos, Facultad de Industrias Alimentarias, Universidad Nacional Agraria La Molina (UNALM), Av. La Molina s/n Lima 12, Lima 15024, Peru; 2 Instituto de Investigación de Bioquímica y Biología Molecular (IIBBM), Universidad Nacional Agraria La Molina (UNALM), Av. La Molina s/n Lima 12, Lima 15024, Peru; 3 Facultad de Ciencias de los Alimentos, Universidad Le Cordon Bleu, Av. General Salaverry 3180, Magdalena del Mar, Lima 15076, Peru; bettit.salva@ulcb.edu.pe

**Keywords:** plant-based meat analogs, D-optimal mixture design, food formulation optimization, desirability function, texture profile analysis, sensory assessment

## Abstract

This study developed a chicken nugget analogue formulated with hydrated textured soy protein (HTSP), a meat analogue based on extruded Sacha Inchi cake (MASI), and hydrated gluten (HWG). Instrumental texture analysis, cooking loss and yield, water activity (a_w_), pH, and color measurements were used to evaluate the effects of these protein sources. An optimal formulation was established using a D-optimal mixture design combined with a desirability function, aiming to maximize the springiness index (0.69) and minimize the hue angle (83.81 °), with a commercial chicken nugget (SF) used as reference. The optimal blend (HTSP 20.75%, MASI 46.25%, and HWG 33%) achieved a desirability value of 0.71 and was experimentally validated within a 95% confidence interval. Physicochemical characterization of the optimal formulation (OF) showed moisture content of 56.04%, protein content of 12.04%, fat content of 4.46%, and carbohydrate content of 25.54%. The OF exhibited low cooking loss (6.82%), high yield (93.18%), and favorable textural properties, including hardness (6.11 N), cohesiveness (0.20), springiness index (0.33), and chewiness index (0.40 N). Color parameters were L* 68.02, a* 1.33, b* 24.35, and a hue angle of 86.87°. Compared with commercial chicken nuggets, the OF showed similar physical and nutritional characteristics but lower fat content. Sensory evaluation using check-all-that-apply (CATA) and hedonic tests (*n* = 70 panelists) indicated that the commercial nugget (SF) had the highest flavor and overall acceptability but was primarily associated with greasy and salty attributes. The OF nugget was positively perceived for its crispiness and breading adhesion, although a slight bitterness was reported. In contrast, the commercial vegan nugget (FF) showed the lowest acceptability due to its dry texture and low juiciness. Overall, the results highlight the potential of plant-based protein blends for developing meat analogues; however, further optimization of flavor and aroma is required. In addition, compliance with regulatory requirements for labeling, allergen disclosure, and novel-ingredient disclosure remains essential for market entry.

## 1. Introduction

According to the Organization for Economic Co-operation and Development (OECD) and the FAO, average global meat consumption in 2023 reached 10.23 kg of chicken, 11.07 kg of pork, and 5.9 kg of beef per capita, while chicken consumption in Latin America reached 23 kg per person [1]. Lifestyle changes have increased the demand for processed foods, such as nuggets, driven by industrialization, marketing, and greater accessibility. Concurrently, growing awareness of healthy eating, human health, and animal welfare has fostered the development of plant-based alternatives to conventional meat products [2]. These analogues aim to replicate key characteristics, including texture, flavor, and physicochemical properties [3]. Commonly used ingredients include soy protein, textured vegetable protein, mushrooms, gluten, cereals, and legumes [4].

Nuggets are semi-processed products that undergo restructuring, breading, and frying to enhance their sensory and structural quality [5]. They are typically manufactured from lower-grade poultry meat and skin, combined with spices, shaped, breaded, fried, and frozen at −18 °C [6,7]. When formulated with non-meat ingredients, such products are classified as meat analogues, designed to mimic the sensory and textural properties of conventional nuggets [8,9]. Soy protein and gluten act as extenders, contributing to comparable texture, appearance, and nutritional profiles [9]. In addition, plant proteins may enhance nutritional value while reducing production costs [4].

Sacha Inchi (*Plukenetia volubilis* L.) cake is a by-product of oil extraction, traditionally used as fertilizer or animal feed. Recently, there has been increased interest in incorporating it into functional foods due to its high protein content and favorable nutritional profile [10,11]. Extrusion processing improves the physicochemical and functional properties of the product, thereby expanding its applicability in products [12]. However, most applications of Sacha Inchi derivatives have been limited to baked products, including flatbreads formulated with pressed cake flour [13], curved cookies (Tuiles) [14], macaron shells as a substitute for almond powder [15], snack-type products [16], and energy bars with Sacha Inchi cake inclusion [17]. In emulsified products such as frankfurter-type sausages, only the oil and pressed cake have been used, rather than the extruded form, which limits their potential as a structural ingredient [18]. Although these by-products provide bioactive compounds suitable for meat product development [18], their application remains restricted. While Sacha Inchi oil has been incorporated into high-moisture meat analogues (HMMA) for lipid enrichment and omega-3 delivery [19,20], the structural functionality of extruded Sacha Inchi cake in products such as nuggets has not yet been investigated. Consequently, despite extensive research on the nutritional and functional properties of Sacha Inchi derivatives [21,22], their integration (in extruded form) into meat analogue systems remains underexplored.

Wheat gluten is primarily composed of gliadins and glutenins, which, upon hydration, form a cross-linked three-dimensional network that generates a fibrous, meat-like structure and confers cohesive and viscoelastic properties [23]. Recent studies have highlighted its role as a key structuring and binding protein in plant-based meat analogues [24]. Modulating the soy protein-to-gluten ratio has been shown to improve fibrous structure, chewiness, and elasticity, underscoring the potential of gluten as both a texture modifier and nutritional enhancer in meat analogues [25,26]. However, studies on plant-based burger analogues indicate that excessive gluten inclusion may impair functional properties, such as water and oil absorption and viscosity, thereby negatively affecting the texture of high-moisture extruded meat analogues [27].

To objectively assess the physicochemical and sensory properties of formulated products, instrumental methodologies commonly used in food science are applied. These approaches enable the objective evaluation of product performance and support formulation development. For formulation optimization, a D-optimal mixture design evaluates the effects of component proportions, constrained to sum to 100%, on selected response variables, enabling predictive modeling and identification of optimal formulations [28,29]. Finally, sensory characterization of the optimized product can be conducted using check-all-that-apply (CATA), a method that identifies attributes relevant to consumer perception [30,31]. Typically, 40–80 participants and up to 30 terms are recommended. CATA has demonstrated effectiveness in meat and health-oriented products, facilitating the identification of consumer-driven sensory attributes [32,33].

Previous studies have shown that plant-based nuggets can achieve acceptable physicochemical performance. However, current formulations still fail to match the sensory attributes of conventional chicken nuggets fully, limiting their competitiveness in the market [34,35,36]. Although plant-based ingredients represent healthier and more sustainable alternatives [4,23,37], the interaction between extruded Sacha Inchi cake and gluten in the formation of elastic or fibrous structures in breaded and fried systems has not been thoroughly investigated. Moreover, the combined application of extruded Sacha Inchi cake and wheat gluten in nugget-type formulations designed to mimic meat-like structure and sensory attributes remains unexplored. While the functional properties of Sacha Inchi derivatives have been characterized individually, their synergistic behavior when combined with gluten and textured soy protein has not been reported. Furthermore, D-optimal mixture designs have not yet been applied to optimize such multi-protein systems in plant-based nugget formulations. Accordingly, applying a mixture design to evaluate a plant-protein-based nugget formulation could provide a framework for advancing the development of viable plant-based alternatives to traditional meat products.

The study seeks to formulate a plant-based nugget using a hydrated textured soy protein (HTSP), a meat analogue based on extruded Sacha Inchi cake (MASI), and hydrated gluten (HWG) as base ingredients, to determine the optimal formulation through a D-optimal mixture design based on physicochemical and textural properties, and to characterize the optimized product using instrumental analyses of texture, color, cooking loss, and yield, as well as sensory evaluation employing the check-all-that-apply (CATA) methodology and a hedonic test.

## 2. Results and Discussion

### 2.1. Proximate and Techno-Functional Analysis of Raw Materials

The proteins used in the mixture design, such as the textured soy protein (TSP), MASI, and wheat gluten (WG), were analyzed for proximate composition, water-holding capacity (WHC), and oil-holding capacity (OHC) (Table 1). Significant differences in moisture content were observed among samples. MASI exhibited the highest moisture level due to its high-moisture extrusion process. In contrast, TSP and WG showed lower moisture levels, attributable to low-moisture extrusion and post-processing drying, respectively. WG showed the highest protein content (76.27%), consistent with its composition, as gluten is mainly composed of gliadins and glutenins, which account for 70–80% of total wheat protein and confer elastic and cohesive properties [38,39]. TSP exhibited a protein content of 60.61%, as it is derived from soy protein concentrate, which typically contains at least 70% protein and is widely used in high-protein extruded products [40].

Significant differences in fat content were observed, although all ingredients had low lipid levels. TSP showed the lowest fat content, consistent with its origin from extruded concentrated soy protein flour, which is inherently low in lipids. MASI also showed low fat levels, as it is derived from Sacha Inchi cake, a by-product of seed oil extraction characterized by high oleic acid content and oil yields ranging from 33.4% to 54.5% [10,41]. Consequently, the resulting cake is a nutritionally valuable agro-industrial by-product, and extrusion has been reported to enhance its functional properties [12,42]. In contrast, WG exhibited the highest fat content among the three ingredients (Table 1). Ash and carbohydrate contents also differed significantly among samples. TSP presented the highest ash (5.31%) and carbohydrate (24.12%) contents. According to the *Codex Alimentarius Commission*, vital wheat gluten should contain at least 80% protein (dry basis), a maximum of 10% moisture, 2% ash, and 1.5% fiber [43]. In comparison, Avellaneda and Pardo [44] reported that extruded Sacha Inchi cake contains 5.6% moisture, 5.83% ash, 4.79% fat, 55.71% protein, 21.47% carbohydrates, and 6.6% fiber.

WHC differed significantly among samples (Table 1), with TSP showing the highest value and WG the lowest. This property is critical for texture and flavor retention in meat analogues, contributing to juiciness and tenderness [45,46]. Soy-based extenders are reported to absorb at least three times their initial weight in water, with WHC values of 4.5–5.0 for soy protein concentrate and up to 5.2 for soy protein isolate [45]. WHC is influenced by protein structure, hydrophilicity, and extrusion conditions, as more porous matrices retain more water [47]. Consistent with this, MASI (high-moisture extrusion) had lower WHC than TSP, which showed the highest value due to its more porous structure [48].

The OHC of TSP and MASI did not differ significantly (Table 1), whereas WG exhibited the highest value (2.35 g oil/dry sample). OHC is associated with the presence of hydrophobic groups on the protein surface and with extrusion effects, which can denature proteins and modify surface hydrophobicity [47]. Higher OHC has been linked to improved palatability in fat-containing products [49]. In addition, insoluble dietary fibers have been reported to enhance OHC and contribute to juiciness and texture, whereas soluble fibers may reduce this capacity [45].

### 2.2. Analysis of the Nugget Formulations

**Figure 1:**
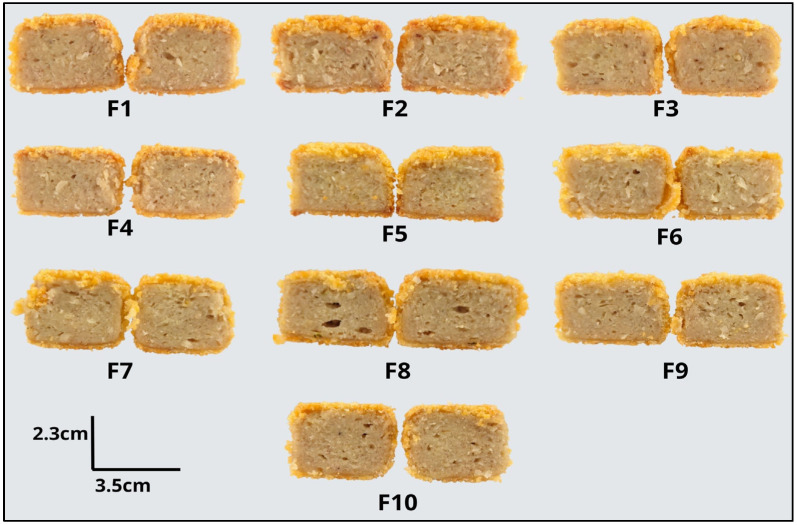
Visual appearance and cross-sectional structure of nugget formulations (F1–F10) produced with different proportions of hydrated textured soy protein (HTSP), meat analogue based on extruded Sacha Inchi cake (MASI), and hydrated wheat gluten (HWG).

Figure 1 presents the visual appearance and cross-sectional structure of the nugget formulations (F1–F10) as influenced by the proportions of HTSP, MASI, and HWG. Differences in matrix compactness, porosity, pore distribution, and structural uniformity can be observed among formulations, suggesting variations in protein composition and network formation within the system. These observations provide a qualitative reference that complements the physicochemical and textural analyses discussed in the following sections.

#### 2.2.1. Analysis of TPA, Cooking Loss, Cooking Yield, a_w_, and pH

Texture profile analysis (TPA) was performed on each nugget substitute formulation containing HTSP, MASI, and HWG, and the following parameters were evaluated: hardness, cohesiveness, springiness index, and chewiness index (Table 2). As reported by Kita et al. [50], texture is a critical quality attribute in fried products. Significant differences in hardness were observed only between formulations F1 and F7 relative to F10, whereas the remaining formulations did not differ significantly. Higher hardness was associated with higher MASI and lower HTSP content, whereas higher HTSP levels were linked to reduced hardness. Accordingly, F10 (20.75% HTSP, 42.44% MASI, 36.81% HWG) exhibited the highest hardness, whereas F7 (62.26% HTSP, 16.98% MASI, 20.76% HWG) showed the lowest. Formulation F3 (32.91% HTSP, 30.28% MASI, 36.81% HWG) exhibited the highest cohesiveness, springiness, and chewiness, whereas F6 (62.26% HTSP, 25.47% MASI, 12.27% HWG) showed the lowest values. Cohesiveness varied only between these two formulations, indicating that the MASI-HWG balance was the primary influence. Springiness and chewiness increased with higher HWG and lower HTSP contents, consistent with the structural role of gluten in enhancing elastic and fibrous networks.

Overall, hardness ranged from 4.86 to 7.47 N, cohesiveness from 0.15 to 0.29, springiness from 0.21 to 0.40, and chewiness from 0.15 to 0.59 N. These parameters are key contributors to perceived tenderness and structural integrity in restructured meat products [51]. Frying-induced protein denaturation and starch gelatinization further modulated these textural attributes [52].

Regarding cooking properties (Table 2), F4 (36.80% HTSP, 50.93% MASI, 12.27% HWG) exhibited the highest cooking loss (9.3%) and the lowest yield (90.8%), whereas F5 (46.21% HTSP, 16.98% MASI, 36.81% HWG) showed the lowest loss (5.8%) and the highest yield (94.2%). The inverse relationship between cooking loss and yield reflects moisture loss during thermal processing [53]. Cao et al. [52] reported that cooking time and temperature significantly affect weight loss and yield; however, in this study, both factors were kept constant. The yields obtained (90.8–94.2%) are consistent with those reported by Akesowan and Jariyawaranugoon [53], who achieved yields between 80.13 and 93% using mushrooms as extenders. Similarly, El-Anany et al. [54] reported yields ranging from 93.4 to 96.6%, attributed to oil absorption during frying.

The water activity (a_w_) ranged from 0.979 to 0.986, with no significant differences among formulations, indicating that variations in the proportions of HTSP, MASI, and HWG did not affect this parameter. Similar a_w_ ranges have been reported for plant-based nuggets formulated with rice bran or chickpea protein [55,56]. These values fall within the range that supports microbial growth (0.70–0.99) [57], highlighting the need for freezing and appropriate packaging to ensure product stability.

The pH values were comparable across formulations (6.61–6.73), in agreement with values reported for other plant-based nuggets [54,58]. Such pH levels are primarily influenced by ingredient composition and thermal processing and may decrease during storage due to microbial activity [59,60].

The analysis of variance (ANOVA) results and model fit indicators (Table 3) showed that the linear regression models for hardness (*p* < 0.0001; R^2^ = 0.66; R^2^ adj = 0.62) and cohesiveness (*p* < 0.0001; R^2^ = 0.30; R^2^ adj = 0.22) exhibited limited explanatory power. In contrast, the models for springiness (*p* < 0.0001; R^2^ = 0.76; R^2^ adj = 0.73) and chewiness (*p* < 0.0001; R^2^ = 0.79; R^2^ adj = 0.77) were statistically significant and exhibited acceptable goodness of fit. The lack-of-fit test supported the springiness model (*p* = 0.1189) but indicated inadequate fit for hardness (*p* = 0.0278), cohesiveness (*p* < 0.0001), and chewiness (*p* = 0.0003). Predicted residual error sum of squares (PRESS) values were low for most responses, except for hardness (6.49), suggesting that linear models were able to describe general trends in the interaction among mixture components.

For technological responses, the models for cooking loss (*p* = 0.0023; R^2^ = 0.36; R^2^ adj = 0.28), cooking yield (*p* = 0.023; R^2^ = 0.36; R^2^ adj = 0.28), and a_w_ (*p* = 0.763; R^2^ = 0.03; R^2^ adj = −0.08) showed low coefficients of determination, indicating limited explanatory power within the mixture space. For this reason, these variables were not considered for modeling and were not included in the final equations (Table 4). In contrast, the pH model (*p* = 0.0049; R^2^ = 0.47; R^2^ adj = 0.40) exhibited a moderate fit, suggesting some dependence on formulation; however, its predictive capability remained limited compared to other responses.

Accordingly, the models corresponding to hardness, springiness, chewiness, and pH were considered for modeling analysis, as they exhibited more consistent behavior within the system, even though some statistical indicators reflected limitations in their explanatory capacity. The selection of responses for modeling was based on an integrated assessment of R^2^ adj–R^2^ pred agreement, lack-of-fit, PRESS, and Adequate Precision (AdPrec), prioritizing variables that showed reproducible trends within the system. This criterion allowed the exclusion of models with low descriptive power and focused the analysis on responses with greater functional relevance.

Response selection for optimization was based on an integrated assessment of R^2^ adj–R^2^ pred agreement, lack-of-fit, PRESS, and Adequate Precision (AdPrec) rather than on R^2^ alone. Among the textural variables, springiness was selected due to its statistical consistency. Although the chewiness index showed a slightly higher R^2^ (0.79 vs. 0.76), springiness exhibited better agreement between R^2^ adj (0.73) and R^2^ pred (0.65), indicating a more parsimonious model with acceptable predictive performance. In contrast, a wide gap between R^2^ and R^2^ pred may suggest overfitting and limited predictive accuracy [29,61,62]. Moreover, the springiness model had a non-significant lack-of-fit (*p* = 0.1189), low PRESS, and AdPrec > 4, all of which support its suitability for prediction [63,64].

Models exhibiting low residual variance and adequate statistical precision can be considered robust even when R^2^ values are moderate, provided that complementary indicators are satisfactory [65,66]. In mixture design, preference is typically given to models with non-significant lack-of-fit and strong functional relevance, particularly when assessing the influence of components on key technological attributes [67]. Within this framework, springiness, a proxy for elasticity and the initial bite of conventional nuggets, was selected as the primary response for optimization based on both statistical adequacy and functional importance.

In this context, low-order (linear) models were employed to preserve the interpretability of component effects within a framework consistent with the expected behavior of the food system. Although higher-order models could improve statistical fit, they may introduce complexity in the interpretation of results and hinder their linkage to real phenomena.

Under this approach, the presence of significant lack of fit in some variables may be attributed to the limited capacity of linear models to fully capture the complexity of the system, which is expected in multicomponent food matrices. Consequently, the models were interpreted as descriptive approximations of system behavior, useful for identifying general trends rather than for strict predictive purposes. Nevertheless, these basic models provide a referential basis for understanding the behavior of the evaluated ingredients, which may be useful for future studies employing these raw materials under experimental conditions that allow for more robust modeling.

Furthermore, the unexplained variability observed in some models may be associated with uncontrolled factors during sample preparation and evaluation, such as mixture heterogeneity, ingredient distribution, processing conditions, and instrumental variations, which are inherent to complex food matrices. These factors are acknowledged as part of the scope of the study and contribute to explaining the behavior observed in variables with lower explanatory power.

The selected models for TPA responses (Table 4) were fitted using linear mixture models to quantify the contribution of each component. MASI exhibited the strongest positive effect on hardness, as reflected by the highest model coefficient. In contrast, HWG exerted the most pronounced positive influence on springiness and chewiness, consistent with its structural role in the formation of elastic and fibrous networks. For pH, similar coefficients across components indicated minimal differential effects of ingredient proportions on these parameters.

**Figure 2:**
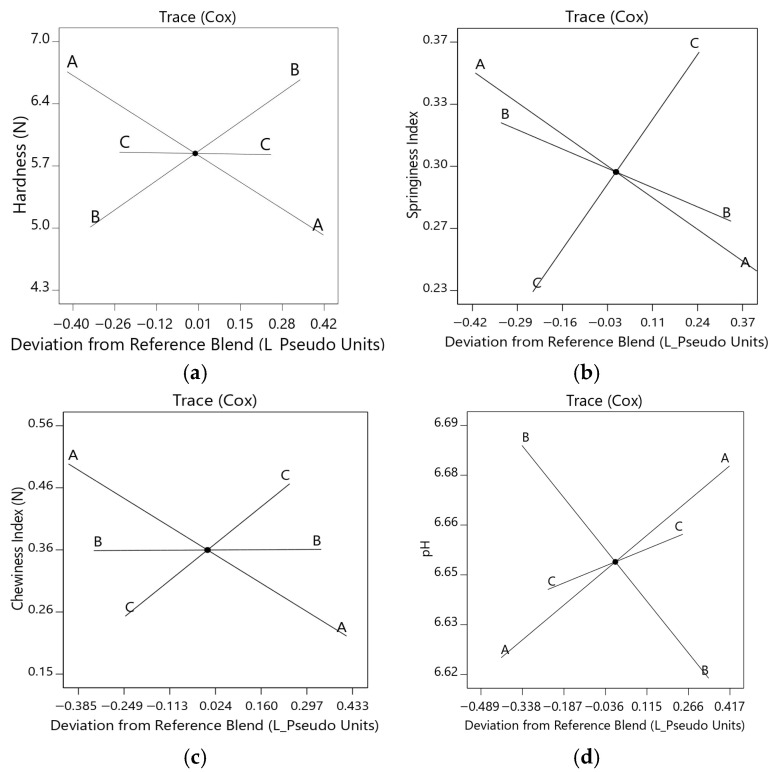
Cox traces illustrating the effect of HTSP (A), MASI (B), HWG (C) on TPA, and pH of nuggets, where HTSP: Hydrated textured soy protein; MASI: Meat analogue based on extruded Sacha Inchi cake; HWG: Hydrated wheat gluten. Also, (**a**): hardness; (**b**): springiness index; (**c**): chewiness index, and (**d**): pH.

The Cox trace plots revealed distinct component effects on textural attributes (Figure 2). The Cox trace plots were interpreted only for the selected responses included in the final modeling (Table 4), as these variables exhibited the most consistent behavior within the mixture system. For hardness (Figure 2a), increasing HTSP decreased hardness, whereas higher MASI increased it, while HWG showed a negligible effect. This trend is consistent with reports indicating that fiber-rich ingredients increase nugget hardness [53,59], whereas hydrated textured proteins reduce chewing force and soften structure [9]. MASI, which is protein- and fiber-rich and not pre-hydrated, therefore contributed to increased hardness at higher proportions.

HWG strongly and positively influenced the springiness response (Figure 2b), while HTSP and MASI showed negative slopes. This reflects the viscoelastic behavior of gluten networks, primarily governed by gliadin and glutenin, which resemble the elastic response of muscle proteins and are widely used for structuring meat analogues [68,69]. For chewiness (Figure 2c), HTSP exhibited an adverse effect, HWG a positive effect, and MASI a minimal influence, as indicated by its near-flat trace. For pH (Figure 2d), the Cox traces showed moderate variations among components, with distinct directional trends. HTSP (A) exhibited a positive slope, indicating an increase in pH with increasing proportion, whereas MASI (B) showed a negative slope, suggesting a decrease in pH. In contrast, HWG (C) presented a slight positive effect, with a lower slope magnitude. Overall, these results indicate that pH was influenced by the relative proportions of the components, although within a relatively narrow range, reflecting a balanced interaction among ingredients rather than a dominant effect of a single component.

Overall, the Cox plots confirmed the trends identified by the selected models, reinforcing the functional role of each component within the mixture system.

The contour plot (Figure S1) presented both two- and three-dimensional surfaces, using five color gradients: blue indicated the lowest values; light blue represented low-to-medium values; green corresponded to medium values; yellow marked the transition to high values; and red indicated the highest values [67]. Notably, the highest hardness values were observed in the green zone (Figure S1a), whereas the lowest were observed in the blue zone. In contrast, the springiness index showed its lowest values in the blue zone (Figure S1c) and the highest in the orange zone. Similarly, chewiness values were lowest in the blue zone and increased progressively toward the green–yellow regions (Figure S1e). In the case of pH, the lowest values were observed in the blue zone, while higher values were distributed toward the green region (Figure S1g).

These graphical representations allow visualization of the relative influence of mixture components on the selected responses, facilitating the interpretation of the trends observed in the modeling.

#### 2.2.2. Instrumental Color

Instrumental color analysis (Table 5) indicated that formulation F2 (49.53% HTSP, 38.20% MASI, 12.27% HWG) exhibited the highest lightness (L*), whereas F10 (20.75% HTSP, 42.44% MASI, 36.81% HWG) showed the lowest, with statistically significant differences. The L* range (67.43–70.14) was comparable to values reported for plant-based and modified nugget systems, suggesting that replacement of chicken with plant proteins primarily affects lightness through non-enzymatic browning during frying [52,54].

Formulation F4 (36.80% HTSP, 50.93% MASI, 12.27% HWG) exhibited the highest a* (red–green), b* (blue–yellow), and chroma (C*), and the lowest hue angle (°h). In contrast, F5 (46.21%, HTSP; 16.98%, MASI; 36.81%, HWG) showed the lowest a* and the highest °h. F7 (62.26%, HTSP; 16.98%, MASI; 20.76%, HWG) had the lowest b* and C* values. Akesowan and Jariyawaranugoon [53] reported a* values between 0.6 and 1.60 and b* values between 13.57 and 17.34 in modified nuggets, while El-Anany et al. [54] reported a* values from 4.5 to 7.74 and b* values from 29.5 to 30.1 in cauliflower-based nuggets. In this study, MASI contributed to higher b* due to its yellow tone, whereas HWG and HTSP had lighter hues. As noted by Moorthi et al. [35], a* and b* are influenced by the pigments present in the ingredients, such as the yellow pigments in chickpeas. Similarly, Polizer et al. [7] emphasized that product color depends on the nature and concentration of these pigments. The C* and °h in the nugget analogues ranged from 23.57 to 24.98 and 86.22° to 88.02°, respectively. Wan et al. [70] reported C* and °h values of 18.42–21.31 and 79.29–80.65° in chicken and oyster mushroom burgers, while Prinyawiwatkul et al. [71] reported wider ranges (C*: 33.2–39.2; °h: 52.7–79.9°) in nuggets enriched with cowpea and peanut flour. Thus, the values in this study fall within or close to previously reported ranges.

ANOVA results and model-fit metrics (Table 6) indicated that the models for L* (*p* = 0.005; R^2^ = 0.46; R^2^ adj = 0.40), b* (*p* = 0.003; R^2^ = 0.69; R^2^ adj = 0.58), and C* (*p* = 0.003; R^2^ = 0.70; R^2^ adj = 0.59) exhibited moderate explanatory power, with L* showing the weakest fit. Despite statistical significance (*p* < 0.05), the relatively large gaps between R^2^, R^2^ adj, and R^2^ pred for L* and C* indicate limited predictive reliability. In contrast, the models for a* (*p* < 0.0001; R^2^ = 0.69; R^2^ adj = 0.66) and °h (*p* < 0.0001; R^2^ = 0.67; R^2^ adj = 0.63) demonstrated better agreement between R^2^, R^2^ adj, and R^2^ pred, supporting their suitability for describing formulation effects on color.

Overall, these results indicate that, although the model has present limitations in predictive capability, they allow the description of general trends in system behavior within the evaluated mixture space, and were therefore considered as descriptive rather than strictly predictive approaches.

Among the color responses, °h was chosen for optimization because it integrates information from both a* and b*, providing a more robust descriptor of the perceived hue. Although its determination coefficients were slightly lower than those of a*, the hue model exhibited greater statistical consistency, evidenced by close agreement between R^2^ pred and R^2^ adj, AdPrec > 4, and low PRESS, indicating a favorable signal-to-noise ratio and internal predictability [29,63,64,72]. Hue also holds functional relevance as it integrates information from both a* and b*, offering a more robust descriptor of overall color, especially important in cooked products [73], where golden, orange, or reddish tones are associated with quality, doneness, and visual appeal, key attributes for consumer acceptance of fried foods like nuggets. Thus, hue was chosen based on its statistical indicators (R^2^, R^2^ adj, R^2^ pred, PRESS, AdPrec) and technological relevance [61]. This decision is also supported by recommendations from the Design-Expert (v9.0.6.2) and authors such as Myers et al. [62], who advocate for prioritizing models with overall consistency, even if their R^2^ values are not the highest.

The predictive models describing the effects of mixture components on L*, a*, b*, C*, and °h (Table 7) were primarily linear, except for b* and C*, which followed quadratic forms. HTSP exerted the strongest influence on L*, whereas MASI was the dominant contributor to a*. HWG showed the greatest positive effect on b*, C*, and °h. Negative interaction terms (HTSP × HWG and MASI × HWG) reduced b* and C*, as reflected by their negative coefficients.

However, these models should be interpreted as simplified representations of the system, since unexplained variability may be associated with uncontrolled factors during processing, such as mixture heterogeneity, ingredient distribution, thermal conditions, and possible instrumental variations, which are inherent to complex food matrices.

**Figure 3:**
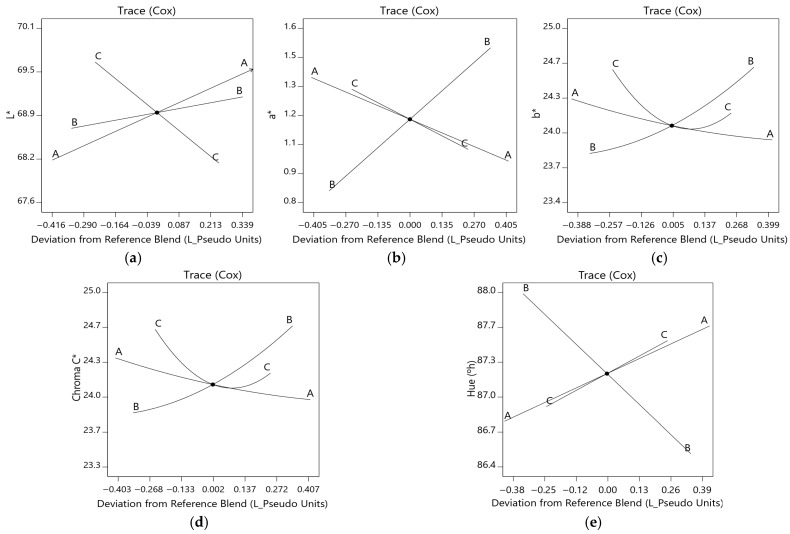
Cox traces illustrating the effect of HTSP (A), MASI (B), and HWG (C) on the color of nuggets. Where HTSP: Hydrated textured soy protein; MASI: Meat analogue based on extruded Sacha Inchi cake; HWG: Hydrated wheat gluten. Also, L*: lightness; a*: green–red coordinate; b*: blue–yellow coordinate; C*: chroma; °h: hue angle, where (**a**), L*; (**b**), a*; (**c**), b*; (**d**), C* and (**e**), °h.

The Cox trace for L* (Figure 3a) indicated that increasing HTSP (A) and MASI (B) increased lightness, with HTSP exhibiting the steepest slope, whereas increasing HWG (C) decreased L*. These trends align with reports attributing nugget lightness to enhanced light reflection by fat–water globules and to the moisture–brightness relationship, where higher moisture increases brightness [74,75]. Accordingly, higher HTSP and MASI levels were associated with increased moisture and greater lightness, whereas HWG reduced brightness due to its inherently darker color.

The a* (Figure 3b) coordinate increased with higher MASI (B) proportions, whereas increasing HTSP (A) and HWG (C) reduced redness. This trend is consistent with reports linking higher redness to lower fat levels in nugget systems [7]. In addition, the reduced occurrence of heme-related reactions in plant-based formulations, such as limited oxymyoglobin formation, has been associated with lower a* values than in meat-based products [34].

The b* (Figure 3c) and C* (Figure 3d) responses showed similar trends: both decreased with increasing HTSP (A), whereas HWG (C) initially declined and then increased. In contrast, increasing MASI (B) raised both b* and C*. Higher b* values correspond to a more yellow appearance [76], consistent with MASI being the most intensely yellow component. Although HTSP also exhibits a yellow hue, its higher lightness and moisture likely reduced color saturation, thereby lowering b* and C* values.

For °h (Figure 3e), increasing HTSP (A) and HWG (C) shifted the tone toward higher °h values (yellowish hues), whereas higher MASI (B) reduced °h. Hue angles between 0° and 90° represent transitions from red to yellow tones [71]. Thus, the interactions among these ingredients modulated the nuggets’ yellow–orange appearance, enhancing or reducing color intensity depending on the combination.

The two- and three-dimensional contour maps (Figure S2) visualized these trends, with maximum L* and a* values located in yellow regions and minima in light-blue zones. For b* and C*, minima occurred in light-blue areas and maxima in orange zones, whereas the highest °h values were concentrated in orange regions.

These graphical representations complement the interpretation of the modeling by enabling visualization of the relative influence of mixture components on color responses, facilitating the identification of general patterns within the system.

### 2.3. Optimization of Response Variables in the Chicken Nugget Substitute and Validation

**Figure 4:**
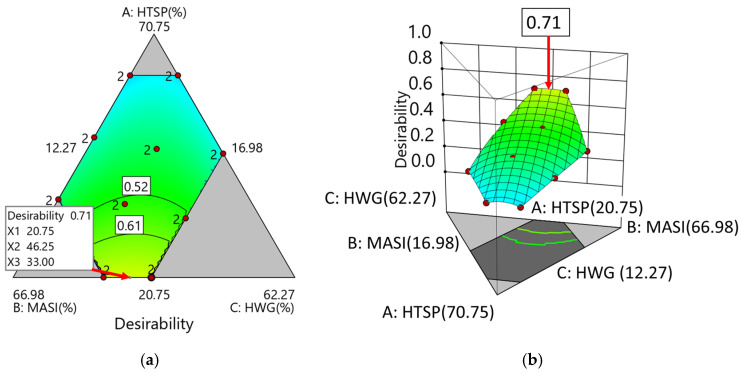
Location of the optimal point on the two-dimensional and three-dimensional contour surfaces: (**a**) 2-D, and (**b**) 3-D. HTSP: Hydrated textured soy protein; MASI: Meat analogue based on extruded Sacha Inchi cake; HWG: Hydrated wheat gluten.

Among three feasible solutions, the formulation with the highest overall desirability (D = 0.71) was selected as optimal, corresponding to 20.75% HTSP, 46.25% MASI, and 33.00% HWG (Table S1). The optimal point was located within the high-desirability region (yellow zone) of both the two-dimensional response surface (Figure 4a) and the three-dimensional desirability map (Figure 4b), indicating a stable optimum within the mixture space.

Simultaneous optimization was performed using a D-optimal mixture design and a desirability function (0–1). Based on model performance, only springiness and hue angle (°h) met the criteria for reliable optimization. This study obtained reference values from a commercial chicken nugget (San Fernando, SF) and implemented constraints in Design-Expert (v9.0.6.2) to guide the solution toward the reference region (Table S2). The numerical optimization aimed to maximize springiness and minimize °h, using reference values of 0.69 and 83.81°, respectively.

To validate the optimal formulation, the predicted and experimental values of the selected responses were statistically compared (Table 8). No significant differences were observed for springiness (0.33) and °h (86.87°), and both values fell within the 95% confidence intervals of the predictions, confirming the model’s adequacy and predictive accuracy as assessed in Design-Expert (v9.0.6.2).

### 2.4. Characterization of the Optimized Chicken Nugget Substitute

#### 2.4.1. Proximate Analysis

The optimized formulation (Table 9) exhibited a moisture content of 56.04%, consistent with a juicier texture, and a protein level of 12.04%, within the range reported for commercial nuggets. The fat content (4.46%) was markedly lower than that of conventional chicken nuggets, indicating a more favorable nutritional profile. Ash content (1.93%) remained low, while carbohydrates (25.54%) contributed to the product’s energy value, and dietary fiber (0.81%) provided a modest functional contribution. Comparable ranges have been reported for commercial chicken nuggets, with moisture (34.71–56.51%), protein (12.52–16.62%), fat (18.14–25%), ash (1.20–1.58%), and carbohydrates (7.52–26.49%) [77], as well as for meat-based formulations showing higher fat and protein levels [59]. In contrast, plant-based nuggets formulated with oyster mushroom and chickpea flour exhibited similar moisture content (55.22–62.90%) but lower protein (6.99–8.74%) and fat (0.47–0.62%) and higher carbohydrate content (26.52–32.71%) [8]. These comparisons indicate that the optimized formulation achieves a balanced nutritional profile between conventional and entirely plant-based nugget systems.

#### 2.4.2. TPA, Cooking Loss, Cooking Yield, a_w_, pH, and Color

Texture Profile Analysis (Table 10) indicated that the optimized formulation (OF) did not differ significantly in hardness from the commercial chicken nugget (SF), although it showed slightly lower values. In contrast, cohesiveness, springiness, and chewiness were significantly lower in the OF nugget. Lukman et al. [77] reported higher ranges of hardness (33.36–77.45 N), cohesiveness (0.61–0.80), springiness (1.00–1.23 mm), and chewiness (23.02–66.13 N·mm^−1^) in commercial chicken nuggets. Similarly, Kitcharoenthawornchai and Harnsilawat [9] reported higher values in meat analog nuggets formulated with texturized vegetable protein (TVP), suggesting that the mechanical properties of the OF nugget were generally lower than those of other chicken-based or analog products.

Cooking loss and yield values of the OF nugget did not differ significantly from those of the SF nugget. This similarity may be attributed to the variability in the industrial processing of the commercial product. At the same time, the OF nugget exhibited greater homogeneity, resulting in slightly lower cooking loss and higher yield. This is beneficial, as lower moisture loss during frying enhances juiciness. Lukman et al. [77] reported cooking losses ranging from 3.37% to 13.05% and yields from 86.95% to 96.63% for commercial chicken nuggets, including the OF nugget. Echeverria et al. [59] highlighted that the inclusion of fibers in plant-based nuggets improves water retention and yield. Husain and Huda-Faujan [8] reported yields exceeding 100% in vegetable nuggets due to oil absorption during frying; however, in the present study, an air fryer was used, which avoided this effect.

The optimal formulation exhibited a high-water activity value (a_w_ = 0.986), significantly higher than that of the commercial reference sample (SF; a_w_ = 0.956), indicating greater availability of free water in the system and potentially lower microbiological stability under uncontrolled conditions. This a_w_ range is compatible with the growth of most spoilage microorganisms and certain foodborne pathogens, representing a limitation in terms of shelf life and food safety [78,79]. However, it is important to consider that the product is intended to be stored under frozen conditions (−18 °C). Under these conditions, a significant fraction of water is present in the solid state, which reduces water availability and limits microbial activity. Nevertheless, this effect is temperature-dependent and reversible upon thawing; therefore, the microbiological stability of the system cannot be attributed solely to reduced water mobility [79].

In this context, product stability should be understood as the result of the combined application of multiple preservation hurdles. Accordingly, the high a_w_ value observed in the optimal formulation suggests the need for additional strategies, such as continuous frozen storage, modified atmosphere packaging, or the incorporation of antimicrobial barriers, such as approved food preservatives, in order to ensure product safety and extend shelf life [80].

Color analysis showed that L* of the OF nugget was significantly lower than that of SF, whereas a*, b*, C*, and °h did not differ significantly (Table 10). The measured ranges are consistent with those reported for chicken nuggets and TVP-based formulations [9,77]. Color differences in fried products are influenced by frying temperature, sample thickness, and heat-induced reactions, including Maillard browning, caramelization, and pigment transformations associated with myoglobin and other proteins [75,81].

### 2.5. Sensory Evaluation

#### 2.5.1. Check All That Apply (CATA)

The CATA test enabled the characterization and differentiation of the sensory profile of three nugget types: OF, mainly composed of HTSP, MASI, and HWG; a commercial vegan nugget (Flex Food, FF); and a commercial chicken nugget (San Fernando, SF). According to Cochran’s Q test, significant differences (*p* < 0.05) were observed for all descriptors except “Cloying” (*p* > 0.05), and the chi-square test confirmed a significant association between products and sensory terms (Tables S3 and S4). Contingency analysis showed that the most frequently selected descriptors were “Crunchy” and “Weak chicken odor” for OF; “Lightly seasoned” and “Weak chicken odor” for FF; and “Easy to chew” and “Soft” for SF (Table S5). For OF, the least selected terms were “Salty”, “Slightly crunchy”, and “Chicken flavor”.

**Figure 5:**
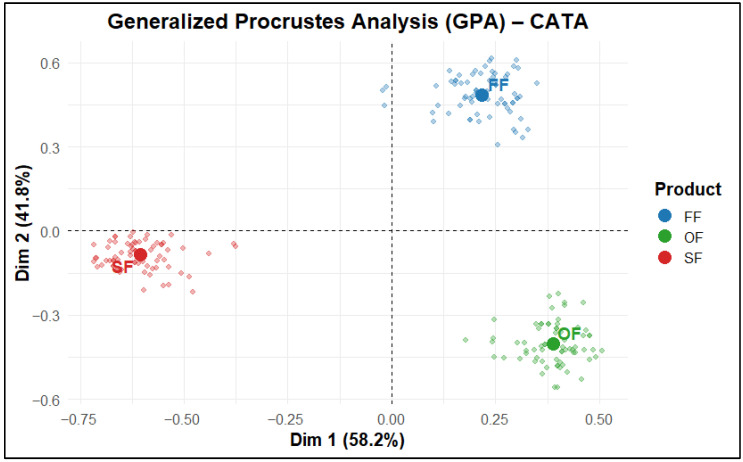
Consensus map from Generalized Procrustes Analysis (GPA), including individual configurations, for the evaluated nuggets: OF (Optimal Formulation), FF (commercial vegan nugget Flex Food), and SF (commercial chicken nugget San Fernando), based on check-all-that-apply (CATA) data.

**Figure 6:**
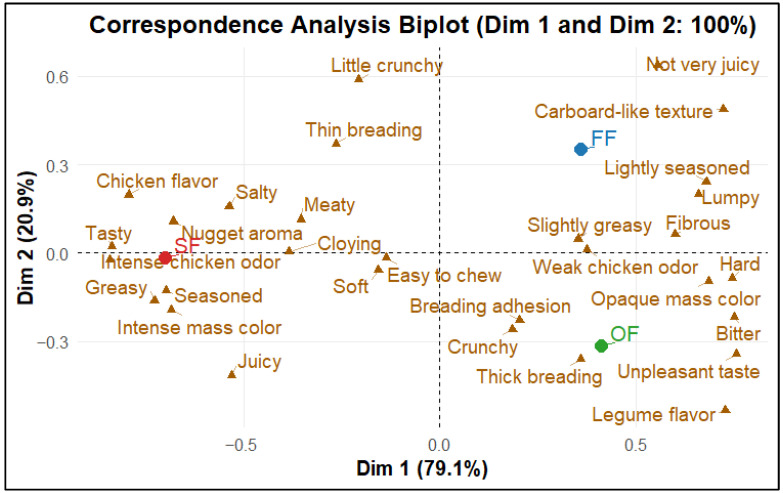
Correspondence Analysis (CA) showing the relationship between nugget samples: OF (Optimal Formulation), FF (commercial vegan nugget Flex Food), and SF (commercial chicken nugget San Fernando), and their associated sensory descriptors based on check-all-that-apply (CATA) data.

The Generalized Procrustes Analysis (GPA) (Figure 5) revealed a clear consensus among panelists and a well-defined separation among the three products, facilitating a unified interpretation of the sensory structure [82]. This differentiation is reinforced by the Correspondence Analysis (CA) biplot (Figure 6), where SF was associated with positive attributes such as “Chicken flavor”, “Juicy”, “Meaty”, “Tasty”, and “Nugget aroma”, consistent with a favorable sensory profile. In contrast, FF was associated with descriptors such as “Cardboard-like texture”, “Not very juicy”, “Lumpy”, “Fibrous”, “Lightly seasoned”, and “Slightly greasy”, which are commonly linked to lower consumer acceptance. OF, in turn, was positioned close to “Thick breading”, “Breading adhesion”, “Crunchy”, “Legume flavor”, “Weak chicken odor”, “Bitter”, “Opaque mass color”, and “Unpleasant taste” (Figure 6). These associations are consistent with the logic of CA, whereby terms located closer to each product are more representative of its perceived sensory profile.

**Figure 7:**
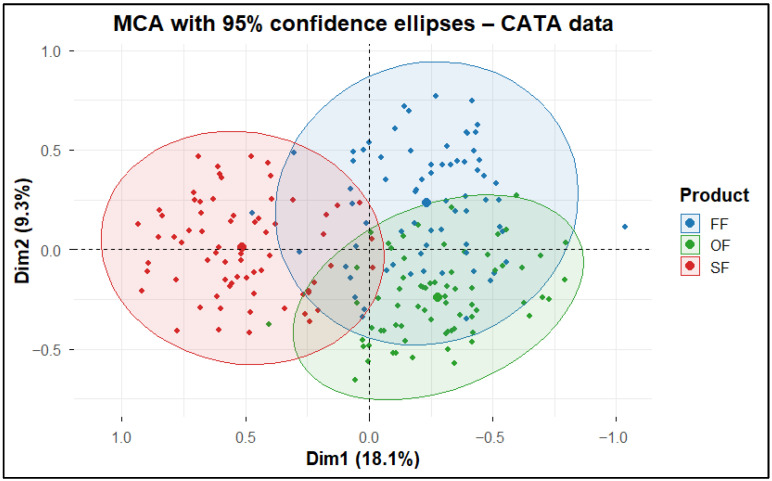
Distribution of consumers and confidence ellipses for the nugget samples: OF (Optimal Formulation), FF (commercial vegan nugget Flex Food), and SF (commercial chicken nugget San Fernando), based on Multiple Correspondence Analysis (MCA) of CATA data.

The Multiple Correspondence Analysis (MCA) with 95% confidence ellipses (Figure 7) showed the dispersion of individual responses using confidence ellipses for each product, indicating a clear separation of SF from the plant-based products, which means that the chicken nugget was perceived as sensorially distinct [83]. Meanwhile, OF and FF exhibited partial overlap, suggesting greater perceptual similarity between the two plant-based products.

**Figure 8:**
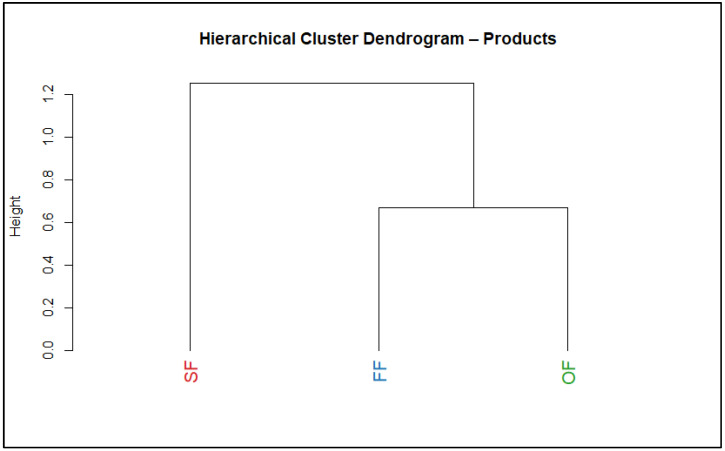
Hierarchical cluster dendrogram derived from CATA sensory descriptors, illustrating the grouping of OF (Optimal Formulation), FF (commercial vegan nugget Flex Food), and SF (commercial chicken nugget San Fernando) according to panelist responses.

The hierarchical cluster dendrogram (Figure 8) confirmed this pattern: OF and FF cluster together, whereas SF forms a separate cluster, highlighting its distinct sensory profile [84].

From an interpretative perspective, the presence of the descriptor ‘Bitter’ in OF may be explained by the use of ingredients such as TSP and MASI, which may be associated with bitter and astringent compounds commonly found in plant-based matrices; in soy-derived ingredients, compounds such as saponins have been reported to contribute to bitterness [85], while in Sacha Inchi by-products, bitterness has been associated with the presence of antinutritional factors [21]; these compounds may not be completely eliminated during processing, depending on the applied thermal conditions, potentially explaining occasional mentions of “Unpleasant taste”. Likewise, descriptors such as “Cardboard-like texture” and “Weak chicken odor” are frequently reported in plant-based products and tend to affect consumer acceptance negatively [86]. Nevertheless, the proximity of OF to breading-related texture attributes such as “Breading adhesion” and “Crunchy” suggests relevant sensory strengths for breaded products. In addition, its distance from “Not very juicy” and “Cloying” may be perceived as an advantage compared with drier or overly cloying profiles.

Finally, the fact that OF is located away from “Salty”, “Seasoned”, and “Greasy” can be discussed as a potentially favorable trait from a nutritional perspective, in line with current trends toward healthier consumption habits [87].

#### 2.5.2. Hedonic Test

The 9-point hedonic test compared Appearance, Color, Odor, Flavor, Texture, and Overall acceptability among OF, FF, and SF (Table 11), using the non-parametric Friedman test with post hoc comparisons. SF exhibited the highest median scores across all attributes, particularly for Flavor, Odor, and Overall acceptability (median = 8), along with narrower interquartile ranges (IQR), indicating greater consistency in consumer preference. In contrast, the plant-based products received lower scores, with FF showing the lowest Overall acceptability (median = 5).

Although SF was rated significantly higher overall, no statistical differences were found between OF and FF for most attributes. This indicates that both plant-based products were perceived as sensorially similar, yet clearly distinct from the traditional chicken nugget. On the hedonic scale, SF was rated as highly pleasant across all attributes, with scores approaching “Like very much”. This favorable perception may be due to its golden, crispy breading, light interior resembling cooked chicken, and a sensory profile enriched by meat ingredients and industrial flavor enhancers. In contrast, the nuggets OF (formulated primarily with HTSP, MASI, and HWG) and the commercial vegan nugget FF received significantly lower ratings. Both plant-based samples scored lower in Appearance, likely due to their dull color and less uniform structure. For Odor and Flavor, they were rated between “Neither like nor dislike” and “Like slightly” respectively, reflecting limited acceptance of plant-based sensory cues. In terms of Texture, SF was rated as “Like moderately,” while OF and FF were rated as “Like slightly”. For Overall Acceptability, SF again received the highest score (“Like very much”), followed by OF (“Like slightly”) and FF (“Neither like nor dislike”).

These results indicated that meat-based formulations, such as the SF nugget, deliver a more favorable sensory experience, likely due to consumers’ familiarity with such products. In contrast, the lower ratings for OF in Odor, Flavor, Texture, and Overall acceptability may stem from the sensory influence of its plant-based ingredients (HTSP, MASI, HWG, among others). Although these are innovative and functional alternatives, they still face the challenge of replicating the sensory traits traditionally associated with chicken nuggets. In this regard, breading and typical meat-based ingredients play a crucial role in perceived juiciness, texture, and flavor, substantially shaping sensory evaluation [88].

**Figure 9:**
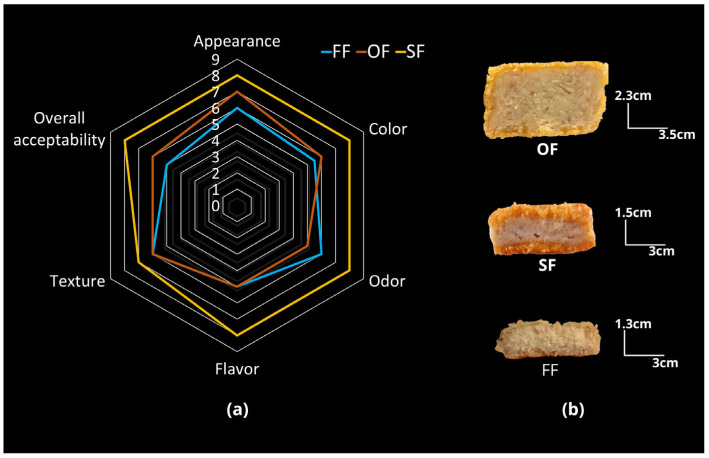
Physical dimensions and hedonic profile of nuggets OF (Optimal Formulation), FF (commercial vegan nugget Flex Food), and SF (commercial chicken nugget San Fernando). (**a**) Radar chart showing median scores for Appearance, Color, Odor, Flavor, Texture, and Overall Acceptability on a 9-point hedonic scale. (**b**) Cross-sectional images with height and width.

As a visual summary, the radar plot (Figure 9a) clearly showed the acceptance hierarchy (SF > OF > FF), consistent with previous reports that conventional meat-based products often outperform plant-based alternatives in aroma, flavor, and texture [86]. The cross-sectional images (Figure 9b) illustrated differences in physical dimensions among products (e.g., greater height in OF, which is suitable for instrumental analyses), which may influence perceptions of texture or juiciness; however, this is presented as a descriptive observation rather than a direct causal relationship.

**Figure 10:**
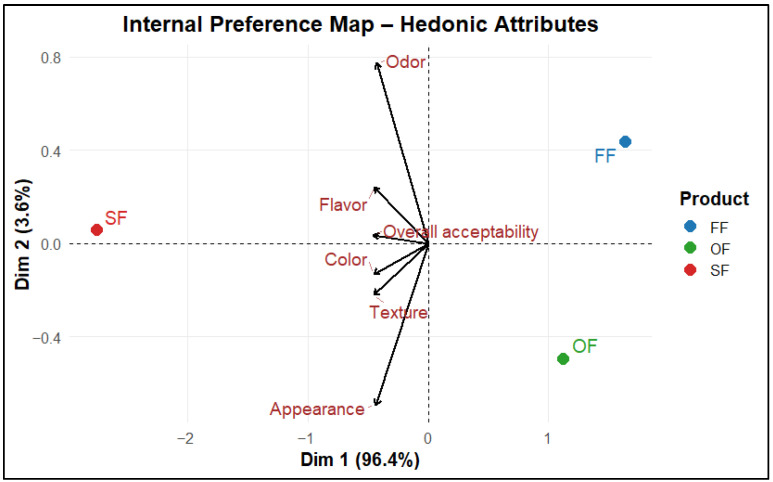
Internal Preference Map (IPM) of hedonic attributes for nuggets OF (Optimal Formulation), FF (commercial vegan nugget Flex Food), and SF (commercial chicken nugget San Fernando). PCA biplot showing the association between products and hedonic attributes (Appearance, Color, Odor, Flavor, Texture, Overall Acceptability).

The Internal Preference Map (IPM) (Figure 10) provided a multivariate interpretation: SF was positioned on the side associated with the hedonic attribute vectors (Dim 1 accounts for 96.4% of the variance), whereas OF and FF are farther away, indicating lower alignment with the dominant preference dimensions.

The observed dispersion suggests greater consensus for SF and higher variability for FF, with OF occupying an intermediate position. Overall, this pattern supports the potential to improve OF, particularly by optimizing aroma and flavor and selected texture elements to better align with consumer preferences [89,90]. These results also reinforce the role of breading, meat-typical ingredients, and aromatic profiles as key determinants influencing consumer perception of nugget-type products [88].

#### 2.5.3. Penalty Analysis: Hedonic Test–CATA

**Figure 11:**
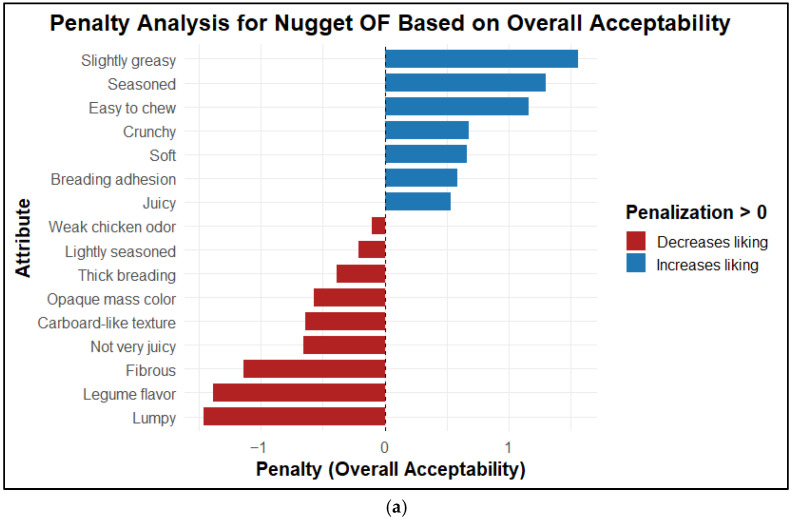

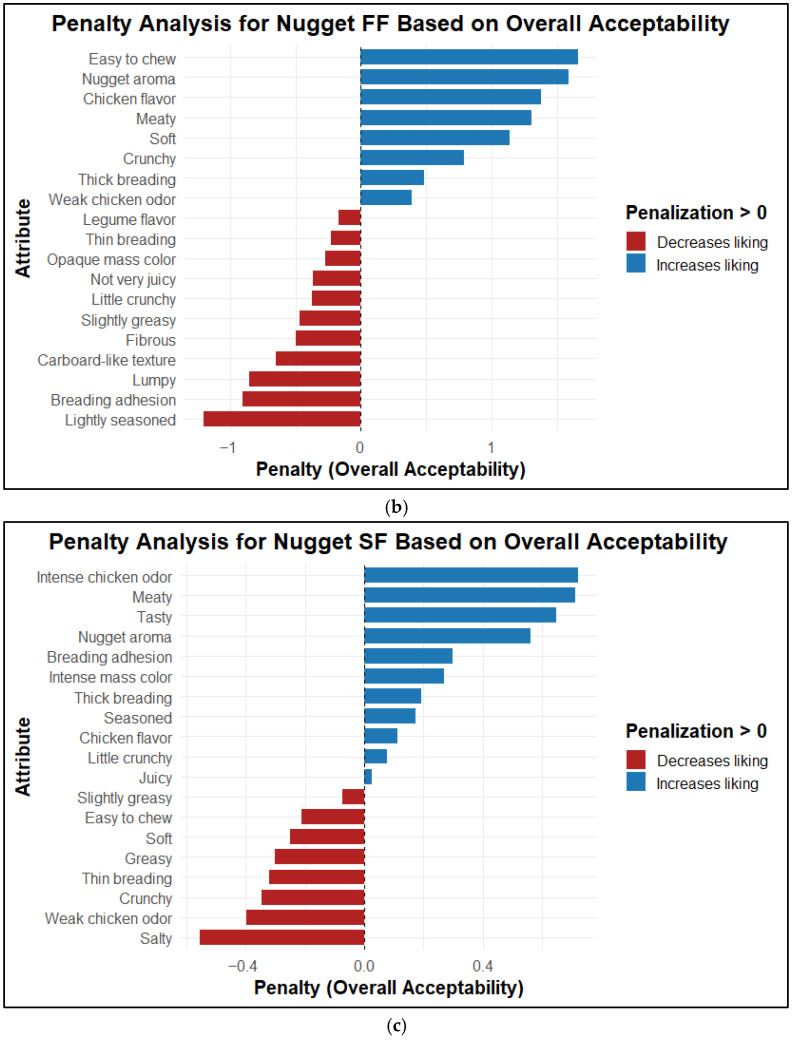
Penalty analysis of sensory attributes influencing the overall acceptability of nuggets: OF: Optimal Formulation; FF: commercial vegan nugget Flex Food; and SF: commercial chicken nugget San Fernando. Where (**a**): Penalty analysis of OF in relation to overall acceptability; (**b**): Penalty analysis of FF in relation to overall acceptability; (**c**): Penalty analysis of SF in relation to overall acceptability. The penalty values (x-axis) represent the decrease in mean acceptability scores from consumer responses.

The penalty analysis (Figure 11) identified the descriptors that, when selected in the CATA test, increased or decreased Overall acceptability by comparing consumer groups that checked or did not check each attribute [91].

For the OF nugget (Figure 11a), the main attributes penalized acceptability was “Lumpy”, “Legume flavor”, “Fibrous”, “Not very juicy”, “Cardboard-like texture”, “Opaque mass color”, “Thick breading”, and “Lightly seasoned”, suggesting that sensory limitations are primarily associated with structural perception, vegetal flavor profile, and low seasoning intensity. In contrast, attributes that increased acceptability were “Slightly greasy”, “Seasoned”, “Easy to chew”, “Crunchy”, “Soft”, “Breading adhesion”, and “Juicy”, highlighting the role of palatability and breading texture in positive product evaluation. For the FF nugget (Figure 11b), the most potent adverse effects were associated with “Lightly seasoned”, “Breading adhesion”, “Lumpy”, “Cardboard-like texture”, “Fibrous”, and “Not very juicy”, whereas “Easy to chew”, “Nugget aroma”, “Chicken flavor”, “Meaty”, “Soft”, and “Crunchy” contributed positively to acceptability, indicating that the main sensory bottlenecks are related to dry or fibrous texture and low flavor intensity. For the SF nugget (Figure 11c), a greater proportion of attributes increased acceptability, such as “Intense chicken odor”, “Meaty”, “Tasty”, “Nugget aroma”, “Breading adhesion”, and “Intense mass color”. In contrast, “Salty”, “Weak chicken odor”, “Crunchy”, “Thin breading”, and “Greasy” acted as deterrents for a subset of consumers, suggesting that even the preferred product may present specific conditions that may reduce acceptance. Overall, this analysis translates sensory perceptions into operational reformulation criteria, enabling the prioritization of critical attributes to improve the acceptability of plant-based nuggets, consistent with previous applications of the CATA–hedonic approach in plant-based product development [90].

### 2.6. Scalability General Panorama and Regulatory Framework

Although this study was conducted at the laboratory scale, a preliminary assessment of its scalability and industrial relevance is warranted. The production of plant-based meat analogues requires precise control of moisture content, temperature, and protein composition to ensure structural stability and processability [92]. As no pilot-scale trials or techno-economic analyses were conducted, the results should be regarded as exploratory for large-scale implementation.

The behavior of gluten and its interaction with other plant proteins are critical during industrial operations such as mixing, molding, and thermal processing. In this study, vital wheat gluten was hydrated and incorporated directly, providing structural functionality through the formation of a viscoelastic network of glutenins and gliadins stabilized by disulfide bonds, which promotes cohesion, elasticity, and water retention [68,93]. Under industrial conditions, enzymes such as transglutaminase may further enhance these functional properties and facilitate performance in continuous production lines [94]. This technological versatility enables the formulation to adapt to different equipment configurations and production scales [95].

Extruded Sacha Inchi cake has demonstrated functional and nutritional benefits in bakery products, snacks, and fortified bars [12,16,96]; however, its performance in structured meat analogues under continuous processing conditions remains limited. Studies using Sacha Inchi oil in high-moisture meat analogues have reported improvements in cooking yield and texture [19,20], reinforcing the need to evaluate the extruded cake in industrial systems. Its use is also aligned with sustainability principles and the valorization of agro-industrial by-products.

From a regulatory perspective, plant-based meat analogues are governed by general food legislation. In the United States, the Food and Drug Administration (FDA) regulates labeling, ingredient safety, and manufacturing practices, and novel ingredients require evaluation under the Generally Recognized as Safe (GRAS) framework [97]. In the European Union, ingredients not significantly consumed before 15 May 1997 fall under Regulation (EU) 2015/2283 on Novel Foods, which mandates safety and nutritional assessments before market entry [97,98]. In Andean countries such as Peru, Sacha Inchi cake is considered a traditional food; however, its incorporation into innovative food matrices may still require additional sanitary authorization [99,100].

In all cases, labeling must clearly declare the plant-based origin and major allergens such as gluten and soy, and nutritional claims must be scientifically substantiated [101,102,103,104].

## 3. Materials and Methods

### 3.1. Materials

The study was conducted at the Universidad Nacional Agraria La Molina (Lima, Peru). All ingredients used for nugget preparation were commercially available in Lima, Peru. These included textured soy protein (RESPON-SE^®^ 4410, Alitecno S.A.C., Lima, Peru), a meat analogue based on extruded Sacha Inchi cake (Agroindustrias Osho S.A.C., Lima, Peru), vital wheat gluten (ViVir^®^, local market, Lima, Perú), gray oyster mushroom (Willka^®^, Lima, Peru), rolled oats (Quaker^®^, Chicago, IL, USA), wheat flour (Nicollini^®^, Lima, Peru), vegetable oil (Bells^®^, Lima, Peru), chicken-flavored seasoning (DoñaGusta^®^, Lima, Peru), iodized salt (Emsal^®^, Lima, Peru), honey (Bells^®^, Lima, Peru), palm vegetable shortening (Palma Tropical^®^, local market, Lima, Peru), methylcellulose (Modernist Pantry^®^, Portsmouth, NH, USA), onion powder (local market, Lima, Peru), sodium tripolyphosphate, soy protein isolate (SUPRO 500E, Alitecno S.A.C., Lima, Peru), carrageenan (GLM Foods S.A.C., Lima, Peru), predust, batter (ARLQ), and breading (BREADER-CR200; Alabama Foods S.A.C., Lima, Peru).

### 3.2. Raw Material Characterization

#### 3.2.1. Proximate Analysis

Moisture, protein, fat, ash, and carbohydrate contents were determined for textured soy protein (TSP), the meat analogue based on extruded Sacha Inchi cake (MASI), and wheat gluten (WG). AOAC (2005) methods were used to analyze the proximate composition of TSP [105]. Methods established by AACC (2009), AOAC (2019), and the Peruvian Technical Standard (NTP) (2017, 2021) guided MASI analysis [106,107,108,109]. AOAC (2019) and NTP guidelines defined the analytical procedures applied to WG [107,110,111,112].

#### 3.2.2. Water and Oil Holding Capacity

The water-holding capacity (WHC) and oil-holding capacity (OHC) of TSP were determined following the method described by Liu et al. [113]. The sample was ground to 18 mesh, and 4 g were transferred to a 50 mL Falcon tube. Subsequently, 30 mL of distilled water or oil was added, and the mixture was homogenized using a vortex mixer at 2000 rpm for 10 min. The samples were centrifuged at 5000× *g* for 20 min, after which the supernatant was discarded, and the precipitate was weighed.

MASI, WHC, and OHC were determined following the method described by Kantanen et al. [48]. The sample was dried at 40 °C and ground using a 0.5 mm mesh. Five grams of the dried sample were weighed and subjected to the same procedure as described for TSP, except that centrifugation was performed at 2000× *g* for 10 min at 6 °C.

The WHC and OHC of WG were determined according to Schopf et al. [39]. Briefly, 0.5 g of the sample was mixed with 10 mL of distilled water or oil in a Falcon tube, vortexed at 2000 rpm for 30 min, and centrifuged at 2000× *g*. WHC and OHC were calculated using Equation (1) and expressed as grams of water or oil per gram of dry sample. In the equation, W_b_ represents the weight after hydration, and W_a_ represents the initial weight before hydration.

(1) $$WHC,OHC=\frac{W_{b}-W_{a}}{W_{a}}$$

### 3.3. Formulation of the Chicken Nugget Substitute

The preparation of the chicken nugget substitute was conducted in sequential stages. First, a pre-emulsion was formulated using palm vegetable shortening (43.48%), water (43.48%), soy protein isolate (8.70%), and carrageenan (4.35%) [55,114,115]. The soy protein isolate was hydrated in water at 90 °C, then carrageenan and vegetable shortening were incorporated. The mixture was homogenized and stored at −18 °C until use [116]. In the second stage, gelatinized oats were prepared by boiling oats in water at a 1:4.5 (*w*/*v*) ratio at 100 °C under continuous stirring for 10 min [55]. TSP was hydrated at a 1:3.78 (*w*/*v*) ratio with added salt (0.32% of the total nugget mass), and wheat gluten was hydrated at a 1:1.5 (*w*/*v*) ratio. Subsequently, the following ingredients were combined and processed in a food processor (RECORD^®^, Lima, Peru) for 3 min: steamed and drained gray oyster mushrooms (6.26%, cooked at 100 °C for 10 min), gelatinized oats (21.29%), pre-emulsion (14.09%), wheat flour (2.50%), chicken-flavored seasoning (1.30%), onion powder (0.63%), methylcellulose (1.93%), and sodium tripolyphosphate (0.32%). HWG was then incorporated and processed for an additional 3 min. Thereafter, HTSP, MASI, and honey (1.56%) were added and mixed for a further 2 min. The resulting dough was refrigerated at 4 °C for 15 min. Then, 15 g portions were shaped and coated through three breading stages: predust, batter (diluted 1:6 *w*/*v* with water), and breading. The nuggets were frozen at −18 °C and cooked in an air fryer (THOMAS^®^, Neunkirchen, Germany) at 200 °C for 10 min before analysis.

### 3.4. Nuggets Analysis

#### 3.4.1. pH

The pH of the nugget samples was determined using a previously calibrated potentiometer (Hanna Instruments^®^, Woonsocket, RI, USA) according to the method described by El-Anany et al. [54]. Briefly, 10 g of raw, unbreaded, thawed nugget mass was homogenized with 50 mL of distilled water. The mixture was filtered through fiber filter paper (0.45 mm pore size), and the pH of the filtrate was measured at room temperature (25 °C).

#### 3.4.2. Instrumental Color

The color of the fried nugget samples was measured at the central region of each sample [55]. A previously calibrated Konica Minolta CR-400 colorimeter (Konica Minolta, Tokyo, Japan) was used to determine the CIELAB color coordinates: lightness (L*), red–green component (a*), and yellow–blue component (b*). Chroma (C*) and hue angle (°h) were calculated using Equations (2) and (3) [117].

(2) $$C^{*}=\sqrt{{\left(a^{*}\right)}^{2}+{\left(b^{*}\right)}^{2}}$$

(3) $${Hue}^{\circ }=\tan^{-1}\left(\frac{a^{*}}{b^{*}}\right)$$

#### 3.4.3. Water Activity (a_w_)

The study measured water activity (a_w_) in raw, unbreaded, and previously thawed nugget dough [55]. Measurements were performed at 25 °C using an AQUALAB Series 3TE water activity meter (Meter Group, Pullman, WA, USA).

#### 3.4.4. Cooking Loss and Cooking Yield

Cooking loss and cooking yield were evaluated by weighing raw and fried nugget samples that were fried at 200 °C for 10 min. Cooking loss was calculated using Equation (4), and cooking yield was calculated using Equation (5) [8].

(4) $$Cooking\;loss\left(\%\right)=\frac{Raw\;weight-Fried\;weight}{Raw\;weight}\times 100\%$$

(5) $$Cooking\;yield\left(\%\right)=\frac{Fried\;weight}{Raw\;weight}\times 100\%$$

#### 3.4.5. Texture Profile Analysis

Texture Profile Analysis (TPA) was conducted using a texturometer (Brookfield^®^ CT3 AMETEK, CTX, Middleborough, MA, USA). The evaluated parameters included hardness (N), cohesiveness, springiness index, and chewiness index (N). The test was performed at 2 mm/s with two compression cycles, a trigger force of 0.05 N, and a 5 kg load cell. Cylindrical samples (1 cm height × 1.5 cm diameter) were obtained from the central portion of the nuggets [60,118].

### 3.5. Experimental Design

#### 3.5.1. Mixture Design

To define the experimental treatments and identify the optimal formulation, a D-Optimal design with vertex constraints was implemented in Design-Expert (v9.0.6.2), requiring that the proportions of components X_1_, X_2_, and X_3_ sum to 100%. These proportions were expressed relative to the total protein phase of the formulation. The variables, such as HTSP (20.75–62.26%), MASI (16.98–50.93%), and HWG (12.27–36.81%), were restricted within defined limits, resulting in ten formulations (Table 12).

The three variable components (HTSP, MASI, and HWG) accounted for 100% of the protein phase of the experimental mixture, while constant proportions of the remaining ingredients were maintained across treatments. Based on these constraints, duplicate formulations (two batches) were prepared for subsequent analyses. Figure S3 shows the distribution of experimental points within the design space.

#### 3.5.2. Model Fitting

A linear mixture model with pairwise interaction terms evaluated the effects of the three mixture components on the quality attributes of the nuggets. The following equation expresses the model:

(6) $$\begin{array}{c} Y_{n}=\beta_{1}\times HTSP+\beta_{2}\times MASI+\beta_{3}\times HWG+\beta_{12}\times HTSP\times MASI+\beta_{13}\times HTSP\times HWG\\ \;\;\;\;\;\;\;+\beta_{23}\times MASI\times HWG \end{array}$$

where Y_n_ represents the response variable for each of the ten formulations, *β*_1_, *β*_2_, and *β*_3_ are the linear regression coefficients associated with HTSP, MASI, and HWG, respectively, and *β*_12_, *β*_13_, and *β*_23_ are the interaction coefficients describing the binary interactions between HTSP-MASI, HTSP-HWG, and MASI-HWG, respectively.

#### 3.5.3. Optimized Formulation

Simultaneous optimization of nugget quality parameters was performed using a desirability function approach. This technique converts each predicted response into a dimensionless desirability value ranging from 0 (completely undesirable) to 1 (entirely desirable), enabling the evaluation of overall performance across multiple responses [29]. Each response variable (Y_n_) was transformed into an individual desirability function (d_n_), and the optimal formulation was identified by maximizing the overall desirability index within the mixture design space [67]. The overall desirability (D) was calculated as the geometric mean of the individual desirability values:

(7) $$D=\sqrt[{n}]{d_{1}\times d_{2}\times \ldots \times d_{n}}$$

where d_1_, d_2_, …, d_n_ represent the individual desirability values of each response variable, “n” is the total number of responses, and D is the overall desirability index [67]. The optimization criteria were defined as maximizing the springiness response (TPA) and minimizing the hue angle (°h), using a commercial chicken nugget as a reference.

#### 3.5.4. Validation and Characterization of the Optimized Formulation

After optimization, the optimal point was identified, providing the specific component proportions and the predicted values for each response based on the fitted mixture models. The study prepared and experimentally evaluated the optimized formulation to validate model performance, and compared the measured and predicted values. The absence of significant differences between predicted and experimental results was used to confirm model adequacy [119]. The optimized nugget formulation was subsequently characterized in terms of proximate composition (moisture, protein, fat, ash, and carbohydrates), TPA, cooking loss, cooking yield, pH, a_w_, and color parameters (L*, a*, b*, C*, and °h).

### 3.6. Sensory Evaluation

The sensory evaluation included 70 untrained consumers, all students from the Universidad Nacional Agraria La Molina (Lima, Peru), aged 18–30 years. The inclusion criteria were habitual consumption of nuggets or analogous products at least twice per month, and the absence of allergies or dietary restrictions related to the product ingredients (soy, gluten, Sacha Inchi, or other components). Exclusion criteria included conditions affecting taste or olfaction (e.g., colds, chronic sinusitis, heavy smoking), as well as participants who did not complete the session or failed to follow instructions.

The sensory evaluation included three nugget types: the optimized formulation (OF), a commercial vegan nugget (Flex Food, FF), and a commercial chicken nugget (San Fernando, SF). Samples were lightly brushed with vegetable oil and cooked in an air fryer at 200 °C for 10 min, then placed in a preheated oven at 45 °C until serving. Before each session, panelists received a brief familiarization (5 min) on the use of the hedonic scale and the CATA questionnaire, with clarification of the instructions. Participants were instructed to rinse their mouths with water between samples to minimize carry-over effects. Each participant received one unit of each nugget type, which the test staff cut in half: one for the CATA test and the other for the hedonic test. The sensory session served the samples in coded containers and presented them in a randomized order.

#### 3.6.1. Check All That Apply (CATA)

Panelists were provided with an evaluation sheet containing 30 sensory terms related to nuggets, along with the coded samples. They were instructed to check all attributes they perceived for each sample, allowing multiple selections. The terms were gathered from the literature on CATA and descriptive analysis of nuggets [46]. The selected descriptors were: easy to chew, fibrous, lumpy, meaty, legume flavor, cloying, lightly seasoned, seasoned, chicken flavor, tasty, unpleasant flavor, cardboard like texture, slightly greasy, greasy, not very juicy, juicy, soft, hard, little crunchy, crunchy, bitter, salty, breading adhesion, thin breaded, thick breading, intense mass color, opaque mass color, nugget aroma, intense chicken odor, weak chicken odor [86,120,121].

#### 3.6.2. Hedonic Test

A 9-point hedonic test was conducted with untrained consumers. Each participant rated their liking for each sample on a scale from 1 (“I dislike it extremely”) to 9 (“I like it extremely”) [122]. The assessed attributes included appearance, color, odor, taste, texture, and overall acceptability.

### 3.7. Statistical Analysis

Each formulation was prepared in two independent batches (batch 1 and batch 2), which were considered as experimental units (*n* = 2). For each batch, multiple technical replicates (≥3 measurements per parameter) were performed, from which a representative mean value was obtained. The final value corresponds to the average of both batches. The coefficients of variation (CV) among replicates were mostly low (generally <15% and in several cases <10%), indicating adequate reproducibility. However, in some specific cases, higher values were observed in variables sensitive to processing, which is expected in complex food systems and may be associated with uncontrolled factors during preparation and measurement, inherent to these types of matrices. Proximate analyses of the raw materials and the optimal formulation were carried out by a specialized external laboratory using standardized methods, with two determinations per sample (*n* = 2). The number of batches evaluated was limited by the availability of raw materials during the experimental development, and this aspect was considered in the interpretation of the results.

The characterization of the raw materials and the optimized product was expressed as the mean ± standard deviation (SD). Mean comparisons were performed using Tukey’s test at a significance level of 5% (α = 0.05). The evaluation of response variables, identification of feasible formulations, and selection of the optimal nugget formulation were conducted using a D-optimal mixture design implemented in Design-Expert (v9.0.6.2). Analysis of variance (ANOVA) was used to assess the significance of the fitted mixture models at the 95% confidence level (α = 0.05). In addition, formulation-dependent measurement variables were analyzed using one-way ANOVA followed by Tukey’s post hoc test (α = 0.05). A Z-test compared the experimental and model-predicted values to validate the optimized formulation at the 95% confidence level (1 − α = 0.95). For sensory data, CATA responses were analyzed using Cochran’s Q test, chi-square test, and symmetry test with XLSTAT (trial version, 2023) and RStudio (R Foundation for Statistical Computing, Vienna, Austria, version 2026.01.1+403). Hedonic data obtained from the 9-point scale were analyzed in RStudio using the non-parametric Friedman test.

## 4. Limitations and Future Research

Among the main strengths of this study is the innovative use of sustainable plant-based ingredients, including extruded Sacha Inchi cake, textured soy protein, and vital wheat gluten, which enabled the valorization of agro-industrial by-products while providing structural functionality in a chicken nugget analogue. The use of a D-optimal mixture design is a robust statistical approach for optimizing ingredient proportions and systematically evaluating their effects on key technological variables such as texture and color. In addition, comprehensive characterization through physicochemical, instrumental, and sensory analyses (CATA and internal preference mapping combined with hedonic testing) provided a multidimensional assessment of product quality. The experimental validation of the optimal formulation, with agreement between predicted and experimental values, further supports the reliability of the developed models.

Regarding limitations, the absence of pilot- or industrial-scale validation restricts direct assessment of technical and economic feasibility under continuous production conditions. No shelf-life or microbiological stability studies were conducted, which are essential to ensure product safety and quality during storage and distribution. Furthermore, detailed amino acid and fatty acid profiling of the ingredients and the final product was not performed, which limited the complete characterization of nutritional value. From an economic perspective, the lack of cost analysis constrained the evaluation of commercial feasibility. Finally, the sensory panel consisted of university students, which may not fully represent the target consumer population and limits the generalizability of acceptance results.

With respect to future research, validation of the formulation under semi-industrial conditions is recommended, including assessment of dough stability during continuous mixing, automated forming, sequential breading, in-line frying, and frozen storage. Comparative cost and energy-efficiency analyses versus conventional chicken nuggets and commercial plant-based analogues are also suggested. Amino acid and fatty acid profiling would help confirm protein quality and the contribution of essential fatty acids, particularly α-linolenic (ω-3) and linoleic (ω-6) acids. Finally, accelerated and real-time shelf-life studies are necessary to evaluate microbiological, physicochemical, and sensory changes during storage and to ensure long-term product quality and safety.

## 5. Conclusions

This study demonstrates the feasibility of developing a plant-based chicken nugget analogue using hydrated textured soy protein (HTSP), meat analogue on extruded Sacha Inchi cake (MASI), and hydrated wheat gluten (HWG), achieving physicochemical and sensory characteristics that approach those of a commercial chicken nugget reference.

The optimal formulation, identified through a D-optimal mixture design, achieved a global desirability of 0.71 and consisted of 20.75% HTSP, 46.25% MASI, and 33% HWG. Experimental validation confirmed the model’s predictive reliability, as no significant differences were observed between predicted and experimental values for the selected response variables.

The optimized product exhibited a nutritional profile comparable to that reported for commercial chicken nuggets in the literature for moisture, protein, and carbohydrate content, while presenting a notably lower fat level. From a technological perspective, the optimal formulation showed reduced cooking loss, higher yield, and acceptable instrumental texture, with no significant difference in hardness relative to the commercial reference. Color attributes were oriented toward yellowish tones, reflecting the intrinsic pigmentation of the plant-based ingredients.

Sensory evaluation using CATA and hedonic testing revealed significant differences in sensory profiles and consumer acceptance among the three nugget types. The commercial chicken nugget (SF) showed superior performance, driven by attributes related to flavor, aroma, and juiciness. The optimized plant-based formulation (OF) and the commercial vegan nugget (FF) were less preferred, particularly in flavor and aroma; however, the OF nugget exhibited favorable textural attributes, such as crunchiness and breading adhesion, indicating greater potential for improvement relative to FF. Penalty analysis highlighted critical sensory drivers of acceptability, underscoring the need to optimize flavor, aroma, and texture further to emulate conventional meat products more closely.

Although the results are promising, additional validation is required to support industrial applications. Future work should include pilot- and semi-industrial-scale trials, shelf-life and microbiological stability studies, and comprehensive nutritional profiling, including amino acid and fatty acid composition. Continued formulation and process optimization to enhance aroma and flavor will be essential to improving consumer acceptance. Overall, this research represents a step toward the development of more sustainable, health-oriented plant-based foods with potential for large-scale, practical production.

## Figures and Tables

**Table 1:** Proximate and techno-functional analysis of TSP, MASI, and WG.

Analysis	TSP	MASI	WG
Moisture (%)	9.85 ± 0.06 ^b^	60.80 ± 0.00 ^a^	8.66 ± 0.03 ^c^
Protein (%)	60.61 ± 0.13 ^b^	26.42 ± 0.04 ^c^	76.27 ± 0.01 ^a^
Fat (%)	0.21 ± 0.10 ^c^	2.50 ± 0.00 ^b^	4.69 ± 0.02 ^a^
Ash (%)	5.31 ± 0.03 ^a^	2.37 ± 0.00 ^b^	0.88 ± 0.01 ^c^
Total carbohydrates (%)	24.12 ± 0.09 ^a^	7.91 ± 0.04 ^c^	9.52 ± 0.01 ^b^
WHC (g H_2_O/g dry sample)	3.78 ± 0.03 ^a^	2.39 ± 0.03 ^b^	1.52 ± 0.15 ^c^
OHC (g oil/g dry sample)	2.13 ± 0.03 ^b^	2.19 ± 0.09 ^b^	2.35 ± 0.03 ^a^

TSP: textured soy protein; MASI: meat analogue based on extruded Sacha Inchi cake; WG: wheat gluten. Data are presented as mean ± SD (*n* = 2) for moisture, protein, fat, ash, and carbohydrate analyses, and as mean ± SD (*n* = 3) for water-holding capacity (WHC) and oil-holding capacity (OHC). Different superscript letters (a–c) within the same row indicate significant differences (*p* < 0.05) according to Tukey’s test.

**Table 2:** Means values of TPA attributes and physicochemical properties of the chicken nugget substitute.

Formulation	Texture				Physicochemical Properties			
	Hardness (N)	Cohesiveness	Springiness Index	Chewiness Index (N)	Cooking Loss (%)	Cooking Yield (%)	a_w_	pH
F1	6.50 ± 0.23 ^ab^	0.20 ± 0.00 ^b^	0.35 ± 0.01 ^ab^	0.45 ± 0.01 ^bc^	8.30 ± 0.7 ^abc^	91.7 ± 0.7 ^bcd^	0.979 ± 0.004 ^a^	6.61 ± 0.01 ^b^
F2	5.74 ± 0.11 ^bc^	0.20 ± 0.00 ^b^	0.23 ± 0.02 ^cd^	0.27 ± 0.02 ^d^	8.60 ± 0.0 ^ab^	91.4 ± 0.0 ^cd^	0.982 ± 0.001 ^a^	6.66 ± 0.05 ^ab^
F3	5.17 ± 0.40 ^bc^	0.29 ± 0.00 ^a^	0.40 ± 0.00 ^a^	0.59 ± 0.05 ^a^	8.70 ± 0.5 ^ab^	91.3 ± 0.5 ^cd^	0.982 ± 0.006 ^a^	6.68 ± 0.01 ^ab^
F4	6.56 ± 0.58 ^ab^	0.20 ± 0.00 ^b^	0.23 ± 0.05 ^cd^	0.30 ± 0.03 ^d^	9.30 ± 0.7 ^a^	90.8 ± 0.7 ^d^	0.981 ± 0.002 ^a^	6.62 ± 0.02 ^b^
F5	5.19 ± 0.35 ^bc^	0.20 ± 0.00 ^b^	0.33 ± 0.04 ^abc^	0.34 ± 0.02 ^d^	5.80 ± 0.2 ^d^	94.2 ± 0.2 ^a^	0.982 ± 0.001 ^a^	6.67 ± 0.02 ^ab^
F6	5.00 ± 0.35 ^c^	0.15 ± 0.03 ^c^	0.21 ± 0.01 ^d^	0.15 ± 0.03 ^e^	5.90 ± 1.1 ^d^	94.1 ± 1.1 ^a^	0.982 ± 0.001 ^a^	6.65 ± 0.03 ^ab^
F7	4.86 ± 0.08 ^c^	0.20 ± 0.00 ^b^	0.29 ± 0.01 ^bcd^	0.28 ± 0.02 ^d^	6.50 ± 0.0 ^bcd^	93.5 ± 0.0 ^abc^	0.983 ± 0.001 ^a^	6.73 ± 0.02 ^a^
F8	5.88 ± 0.41 ^bc^	0.20 ± 0.00 ^b^	0.31 ± 0.01 ^abcd^	0.36 ± 0.01 ^cd^	6.10 ± 0.2 ^cd^	93.9 ± 0.2 ^ab^	0.982 ± 0.001 ^a^	6.63 ± 0.02 ^b^
F9	5.86 ± 0.12 ^bc^	0.20 ± 0.00 ^b^	0.31 ± 0.01 ^abcd^	0.36 ± 0.00 ^cd^	6.00 ± 0.1 ^cd^	94.0 ± 0.1 ^ab^	0.986 ± 0.000 ^a^	6.67 ± 0.01 ^ab^
F10	7.47 ± 0.52 ^a^	0.20 ± 0.00 ^b^	0.33 ± 0.05 ^abc^	0.49 ± 0.03 ^b^	6.50 ± 0.9 ^bcd^	93.5 ± 0.9 ^abc^	0.983 ± 0.000 ^a^	6.62 ± 0.01 ^b^

a_w_: Water activity. Data are presented as mean ± SD (standard deviation) (*n* = 2 independent batches, with at least three technical replicates per batch). Different superscript letters (a–e) within a column indicate significant differences (*p* < 0.05) using Tukey’s test.

**Table 3:** ANOVA, fitting quality of polynomial models for TPA, cooking loss, cooking yield, a_w_, and pH.

Dependent Variables	ANOVA						Fitting Quality					
	Model	Linear Mixture Terms	HTSP × MASI	HTSP × HWG	MASI × HWG	Lack of Fit	CV (%)	PRESS	R^2^	R^2^ adj	R^2^ pred	AdPrec
Hardness (N)	16.39 (0.0001)	16.39 (0.0001)	-	-	-	3.82 (0.028)	8.99	6.49	0.66	0.62	0.52	10.35
Cohesiveness	3.61 (0.05)	3.61 (0.05)	-	-	-	18.06 (<0.0001)	14.42	0.02	0.30	0.22	0.02	4.43
Springiness index	27.19 (<0.0001)	27.19 (<0.0001)	-	-	-	2.25 (0.12)	10.55	0.02	0.76	0.73	0.65	11.59
Chewiness index (N)	32.88 (<0.0001)	32.88 (<0.0001)	-	-	-	12.61 (0.0003)	16.41	0.08	0.79	0.77	0.71	14.51
Cooking loss (%)	4.77 (0.023)	4.77 (0.023)	-	-	-	8.73 (0.001)	16.37	31.31	0.36	0.28	0.14	5.81
Cooking yield (%)	4.77 (0.023)	4.77 (0.023)	-	-	-	8.73 (0.001)	1.26	31.31	0.36	0.28	0.14	5.81
a_w_	0.2743 (0.763)	0.2743 (0.763)	-	-	-	1.01 (0.479)	0.25	0.0001	0.03	−0.08	−0.30	1.47
pH	7.39 (0.005)	7.39 (0.005)	-	-	-	2.74 (0.072)	0.45	0.02	0.47	0.40	0.28	6.66

F-test and *p* (in parentheses, Prob > F) of the full model. “-”, term eliminated from the equation because it is not significant (*p* > 0.05). HTSP: Hydrated textured soy protein; MASI: Meat analogue based on extruded Sacha Inchi cake; HWG: Hydrated wheat gluten. Measurements of goodness-of-fit were as follows: coefficient of variation (CV), predicted residual error sum of squares (PRESS), coefficient of determination (R^2^), R^2^ adjusted (R^2^ adj), R^2^ predicted (R^2^ pred), and adequate precision (AdPrec.).

**Table 4:** Polynomial models predicted in the analysis of TPA, and pH.

Dependent Variables	Predicted Model Equations *
Hardness (N)	Y_1_ = 3.31HTSP + 8.94MASI + 5.74HWG
Springiness index	Y_3_ = 0.15HTSP + 0.20MASI + 0.69HWG
Chewiness index (N)	Y_4_ = −0.041HTSP + 0.363MASI + 1.030HWG
pH	Y_8_ = 6.73HTSP + 6.51MASI + 6.70HWG

HTSP: Hydrated textured soy protein; MASI: Meat analogue based on extruded Sacha Inchi cake; HWG: Hydrated wheat gluten. * Only the most statistically significant and consistent models are presented (hardness, springiness index, chewiness index, and pH). Variables that were not statistically significant and showed low predictive performance (cooking loss, cooking yield, and a_w_) were not included.

**Table 5:** Mean values of the color parameters L*, a*, b*, C*, and °hue of the chicken nugget substitute.

Formulation	Color Parameters				
	L*	a*	b*	C*	°h
F1	68.25 ± 0.88 ^ab^	1.37 ± 0.01 ^abc^	24.44 ± 0.13 ^ab^	24.48 ± 0.13 ^ab^	86.79 ± 0.00 ^cde^
F2	70.14 ± 0.53 ^a^	1.25 ± 0.03 ^abcd^	24.49 ± 0.13 ^ab^	24.52 ± 0.13 ^ab^	87.09 ± 0.08 ^abcde^
F3	69.37 ± 0.25 ^ab^	0.92 ± 0.25 ^cd^	24.08 ± 0.33 ^ab^	24.10 ± 0.33 ^ab^	87.82 ± 0.55 ^abc^
F4	69.95 ± 0.02 ^a^	1.65 ± 0.10 ^a^	24.92 ± 0.18 ^a^	24.98 ± 0.19 ^a^	86.22 ± 0.19 ^e^
F5	68.18 ± 0.02 ^ab^	0.84 ± 0.14 ^d^	24.19 ± 0.69 ^ab^	24.21 ± 0.70 ^ab^	88.02 ± 0.26 ^a^
F6	69.33 ± 0.93 ^ab^	0.89 ± 0.04 ^d^	24.37 ± 0.08 ^ab^	24.38 ± 0.08 ^ab^	87.90 ± 0.10 ^ab^
F7	69.14 ± 0.19 ^ab^	0.94 ± 0.18 ^bcd^	23.55 ± 0.13 ^b^	23.57 ± 0.13 ^b^	87.74 ± 0.42 ^abcd^
F8	69.20 ± 0.74 ^ab^	1.30 ± 0.09 ^abcd^	24.00 ± 0.09 ^ab^	24.03 ± 0.09 ^ab^	86.91 ± 0.21 ^bcde^
F9	68.21 ± 0.09 ^ab^	1.21 ± 0.09 ^abcd^	24.30 ± 0.03 ^ab^	24.33 ± 0.03 ^ab^	87.16 ± 0.22 ^abcde^
F10	67.43 ± 0.21 ^b^	1.40 ± 0.06 ^ab^	24.38 ± 0.08 ^ab^	24.42 ± 0.08 ^ab^	86.73 ± 0.13 ^de^

L*: lightness; a*: green–red coordinate; b*: blue–yellow coordinate; C*: chroma; °h: hue angle. Data are presented as mean ± SD (*n* = 2 independent batches, with at least three technical replicates per batch). Different superscript letters (a–e) within a column indicate significant differences (*p* < 0.05) using Tukey’s test.

**Table 6:** ANOVA, fitting quality of polynomial models for color parameters.

Dependent Variables	ANOVA						Fitting Quality					
	Model	Linear Mixture Terms	HTSP × MASI	HTSP × HWG	MASI × HWG	Lack-of-Fit	CV %	PRESS	R^2^	R^2^ adj	R^2^ pred	AdPrec
L*	7.36 (0.005)	7.36 (0.005)	-	-	-	3.25 (0.045)	1.04	11.75	0.46	0.40	0.27	6.34
a*	19.24 (<0.0001)	19.24 (<0.0001)	-	-	-	3.06 (0.054)	13.74	0.60	0.69	0.66	0.59	11.22
b*	6.21 (0.003)	11.02 (0.001)	0.0018 (0.97)	4.05 (0.064)	6.55 (0.023)	0.891 (0.504)	1.06	2.22	0.69	0.58	0.26	8.69
Croma C*	6.48 (0.003)	11.73 (0.001)	0.0010 (0.98)	3.90 (0.068)	6.49 (0.023)	0.859 (0.520)	1.07	2.25	0.70	0.59	0.28	8.86
Hue (°h)	17.40 (<0.0001)	17.40 (<0.0001)	-	-	-	3.40 (0.040)	0.43	3.19	0.67	0.63	0.56	10.60

L*: lightness; a*: green–red coordinate; b*: blue–yellow coordinate. HTSP: Hydrated textured soy protein; MASI: Meat analogue based on extruded Sacha Inchi cake and HWG: Hydrated wheat gluten. F-test and *p* (in parentheses, Prob > F) of the full model. “-”, term eliminated from the equation because it is not significant (*p* > 0.05). Measurements of goodness-of-fit were as follows: coefficient of variation (CV), predicted residual error sum of squares (PRESS), coefficient of determination (R^2^), R^2^ adjusted (R^2^ adj), R^2^ predicted (R^2^ pred), and adequate precision (AdPrec).

**Table 7:** Polynomial models predicted in the color analysis corresponding to L*, a*, b*, C*, and °h in the nuggets.

Dependent Variables	Predicted Model Equations
L*	Y_7_ = 70.85HTSP + 69.80MASI + 64.43HWG
a*	Y_8_ = 0.63HTSP + 2.47MASI + 0.31HWG
b*	Y_9_ = 20.01HTSP + 27.85MASI + 35.06HWG + 0.22HTSP × MASI − 18.26HTSP × HWG − 25.30MASI × HWG
C*	Y_10_ = 23.99HTSP + 27.98MASI + 34.99HWG + 0.16HTSP × MASI − 18.05HTSP × HWG − 25.35MASI × HWG
°h	Y_11_ = 88.47HTSP + 84.39MASI + 89.10HWG

L*: lightness; a*: green–red coordinate; b*: blue–yellow coordinate; C*: chroma; °h: hue angle. HTSP: Hydrated textured soy protein; MASI: Meat analogue based on extruded Sacha Inchi cake; HWG: Hydrated wheat gluten.

**Table 8:** Statistical validation of the predictive and actual values of the response variables considered in the optimal formulation.

Response Variable	Predictive Value	Minimum Predictive Value	Maximum Predictive Value	Experimental Value *
Springiness index	0.35 ^a^	0.30	0.40	0.33 ± 0.01 ^a^
Hue (°h)	86.79 ^b^	86.15	87.43	86.87 ± 0.18 ^b^

* Data are expressed as means (*n* = 2 independent batches, with at least three technical replicates per batch). Experimental value ± SD and predictive values with the minimum and maximum confidence limits of the predictive values at 95% confidence. Mean values with the same superscript letters in the same row indicate a non-significant difference (*α* > 0.05).

**Table 9:** Proximate analysis of the optimized chicken nugget substitute.

Analysis (%)	Optimal Formulation
Moisture	56.04 ± 0.06
Protein	12.04 ± 0.01
Fat	4.46 ± 0.04
Ash	1.93 ± 0.05
Total carbohydrates	25.54 ± 0.02
Fiber	0.81 ± 0.01

Data are presented as mean ± SD (*n* = 2).

**Table 10:** TPA, cooking loss, cooking yield, a_w_, pH, and color.

Analysis	Optimal Formulation	San Fernando
Hardness (N)	6.11 ± 0.88 ^a^	8.11 ± 0.98 ^a^
Cohesiveness	0.20 ± 0.00 ^b^	0.31 ± 0.02 ^a^
Springiness index	0.33 ± 0.01 ^b^	0.69 ± 0.02 ^a^
Chewiness index (N)	0.40 ± 0.05 ^b^	1.74 ± 0.05 ^a^
Cooking loss (%)	6.82 ± 0.32 ^a^	19.42 ± 6.14 ^a^
Cooking yield (%)	93.18 ± 0.32 ^a^	80.58 ± 6.14 ^a^
a_w_	0.986 ± 0.000 ^a^	0.956 ± 0.000 ^b^
pH	6.62 ± 0.01 ^b^	6.83 ± 0.01 ^a^
L*	68.02 ± 0.08 ^b^	69.20 ± 0.13 ^a^
a*	1.33 ± 0.06 ^a^	2.04 ± 0.69 ^a^
b*	24.35 ± 0.25 ^a^	19.31 ± 1.97 ^a^
C*	24.38 ± 0.24 ^a^	19.43 ± 1.88 ^a^
°h	86.87 ± 0.18 ^a^	83.81 ± 2.68 ^a^

Data are presented as mean ± SD (*n* = 2 independent batches, with at least three technical replicates per batch). L*: lightness; a*: green–red coordinate; b*: blue–yellow coordinate; C*: chroma and °h: hue angle. Mean values with different superscript letters in the same row indicate a significant difference (*p* < 0.05).

**Table 11:** Median of the liking scores of six sensory attributes for the three types of samples: Flex Food vegan nuggets, optimal formulation, and San Fernando chicken nuggets.

Attributes	OF	FF	SF
	Median IQR	Median IQR	Median IQR
Appearance	7 [5–8] ^ab^	6 [5–7] ^b^	8 [7–9] ^a^
Color	6 [5–7] ^b^	5.5 [4–7] ^b^	8 [7–9] ^a^
Odor	5 [4–6.25] ^b^	6 [4.75–7] ^ab^	8 [6–8] ^a^
Flavor	5 [3–7] ^b^	5 [4–6.25] ^b^	8 [8–9] ^a^
Texture	6 [5–7] ^a^	6 [5–7] ^a^	7 [6–8] ^a^
Overall acceptability	6 [4–7] ^b^	5 [4–7] ^b^	8 [7–8] ^a^

Values in brackets are the interquartile range (IQR) from quartile 1 to quartile 3. Data are expressed as median and interquartile range (IQR) brackets. Values with different superscripts in the same row indicate a significant difference. OF: Optimal Formulation; FF: commercial vegan nugget Flex Food; and SF: commercial chicken nugget San Fernando.

**Table 12:** Formulations for the chicken nugget substitute.

Formulations	HTSP (%)	MASI (%)	HWG (%)
F1	20.75	50.93	28.32
F2	49.53	38.20	12.27
F3	32.91	30.28	36.81
F4	36.80	50.93	12.27
F5	46.21	16.98	36.81
F6	62.26	25.47	12.27
F7	62.26	16.98	20.76
F8	47.16	28.30	24.54
F9	35.84	39.62	24.54
F10	20.75	42.44	36.81

HTSP: Hydrated textured soy protein; MASI: Meat analogue based on extruded Sacha Inchi cake; HWG: Hydrated wheat gluten.

## Data Availability

The data presented in this study are available upon request from the corresponding authors. The data are not publicly available due to privacy concerns.

## Supplementary Materials

The following supporting information can be downloaded at: https://www.mdpi.com/article/10.3390/molecules31101601/s1, Figure S1: Two-dimensional contour surface (2-D, left) and three-dimensional contour surface (3-D, right) plots showing the effect of hydrated textured soy protein (HTSP, A), meat analogue based on extruded Sacha Inchi cake (MASI, B), and hydrated wheat gluten (HWG, C) on the texture profile analysis (TPA) attributes, and pH of the meat analogue nugget formulations, where: (a), hardness 2-D; (b), hardness 3-D; (c), springiness index 2-D; (d), springiness index 3-D; (e), chewiness index 2-D; (f), chewiness index 3-D; (g), pH 2-D; and (h), pH 3-D; Figure S2: Two-dimensional contour surface (2-D, left) and three-dimensional surface (3-D, right) plots showing the effect of hydrated textured soy protein (HTSP, A), meat analogue based on extruded Sacha Inchi cake (MASI, B), and hydrated wheat gluten (HWG, C) on the color properties of the meat analogue nugget formulations, where: (a), L* (Lightness) 2-D; (b), L* (Lightness) 3-D; (c), a* (green–red) 2-D; (d), a* (green–red) 3-D; (e), b* (blue–yellow) 2-D; (f), b* (blue–yellow) 3-D; (g), C* (Chroma) 2-D; (h), C* (Chroma) 3-D; (i), °h (Hue angle) 2-D; and (j), °h (Hue angle) 3-D; Figure S3: Experimental points within the experimental region of the constrained mixture design. HTSP: Hydrated textured soy protein; MASI: Meat analogue based on extruded Sacha Inchi cake; HWG: Hydrated wheat gluten. “2”: The experiment was conducted in duplicate; Table S1: Feasible formulations and desirability values; Table S2: Variable Adjustment Limits for Optimization; Table S3: Cochran’s Q test for each attribute in the nuggets; Table S4: Test of independence between rows and columns in CATA analysis; Table S5: Contingency values of the terms created from the CATA data.

## Abbreviations

The following abbreviations are used in this manuscript:

HTSP	Hydrated textured soy protein
MASI	Meat analogue based on extruded Sacha Inchi cake
HWG	Hydrated wheat gluten
a_w_	Water activity
OF	Optimal formulation
FF	Commercial vegan nugget Flex Food
SF	Commercial chicken nugget San Fernando
CATA	Check-all-that-apply
HMMA	High-moisture meat analogues
TPA	Texture Profile Analysis
L*	Lightness
a*	Green–red
b*	Blue–yellow
C*	Chroma
°h	Hue angle
TSP	Textured soy protein
WG	Wheat gluten
GPA	Generalized Procrustes Analysis
WHC	Water-holding capacity
OHC	Oil-holding capacity
ANOVA	Analysis of variance
CV	Coefficient of variation
SD	Standard deviation
PRESS	Predicted residual error sum of squares
R^2^	Coefficient of determination
R^2^ adj	Adjusted coefficient of determination
R^2^ pred	Predicted coefficient of determination
AdPrec	Adequate precision
CA	Correspondence analysis
MCA	Multiple correspondence analysis
IQR	Interquartile range
IPM	Internal preference map
PCA	Principal component analysis
