# Supplementary material for: Development and Characterization of a Plant-Based Chicken Nugget Analogue Based on Extruded Sacha Inchi Cake, Textured Soy Protein, and Wheat Gluten
Source: Molecules. 2026 May 10;31(10):1601. doi: 10.3390/molecules31101601 (PMC13209919; doi:10.3390/molecules31101601)
Supplement: Supplementary file 1 [file molecules-31-01601-s001.zip › molecules-4255072-supplementary.pdf]

Supplementary Materials

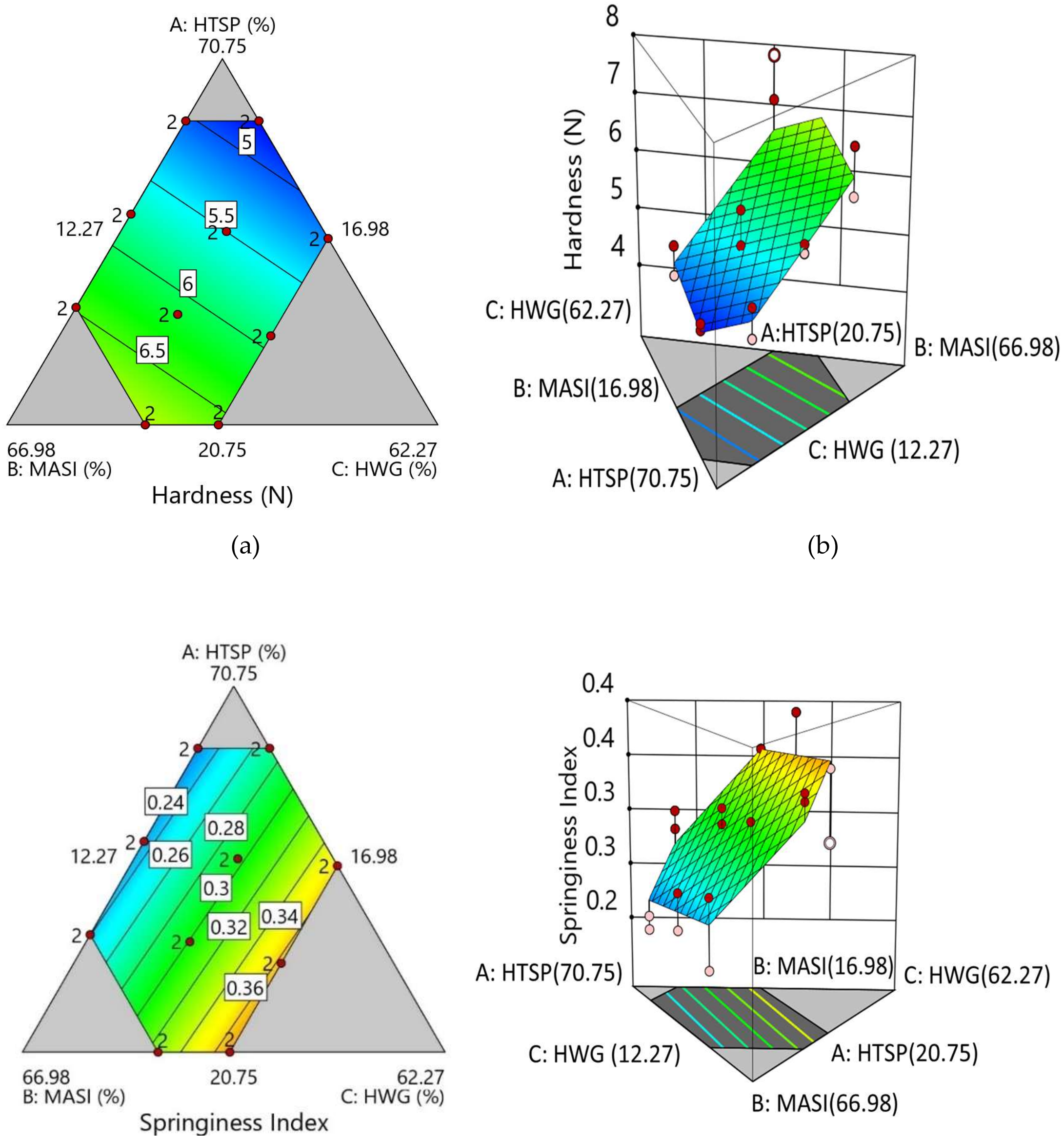

(a) (b)

(c) (d)

Figure S1. *Cont.*

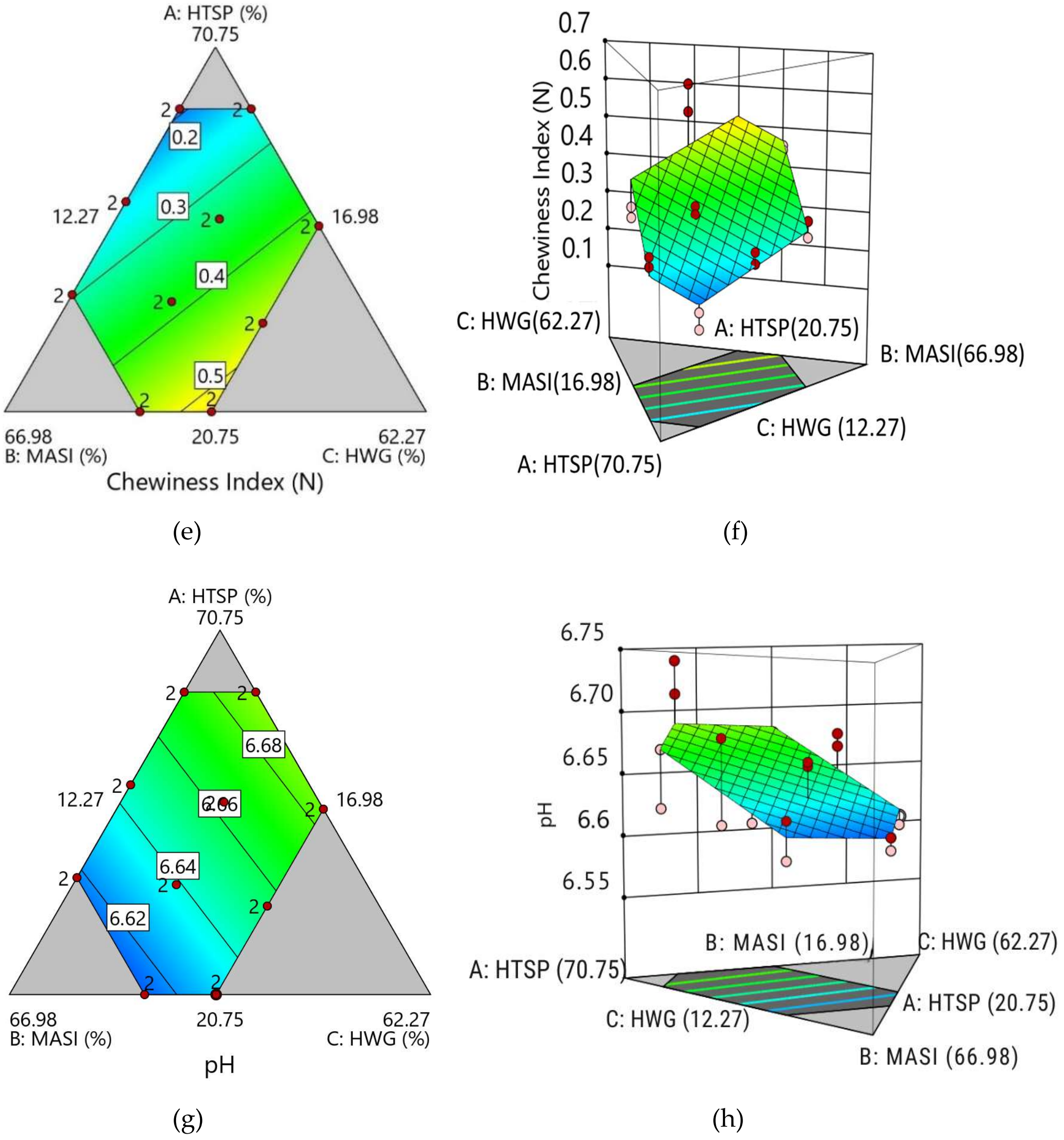

Figure S1: Two-dimensional contour surface (2-D, left) and three-dimensional contour surface (3-D, right) plots showing the effect of hydrated textured soy protein (HTSP, A), meat analogue based on extruded Sacha Inchi cake (MASI, B), and hydrated wheat gluten (HWG, C) on the texture profile analysis (TPA) attributes, and pH of the meat analogue nugget formulations, where: (a), hardness 2-D; (b), hardness 3-D; (c), springiness index 2-D; (d), springiness index 3-D; (e), chewiness index 2-D; (f), chewiness index 3-D; (g), pH 2-D; and (h), pH 3-D.

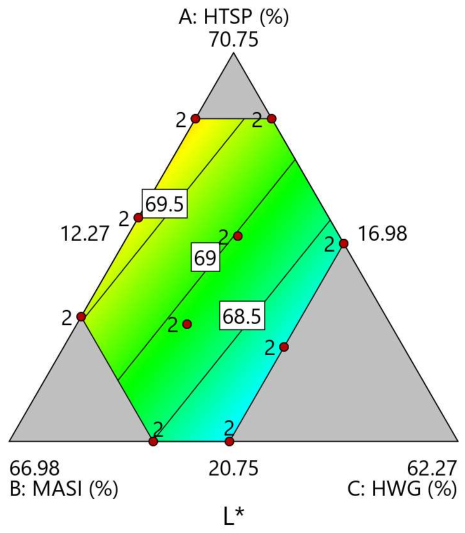

(a)

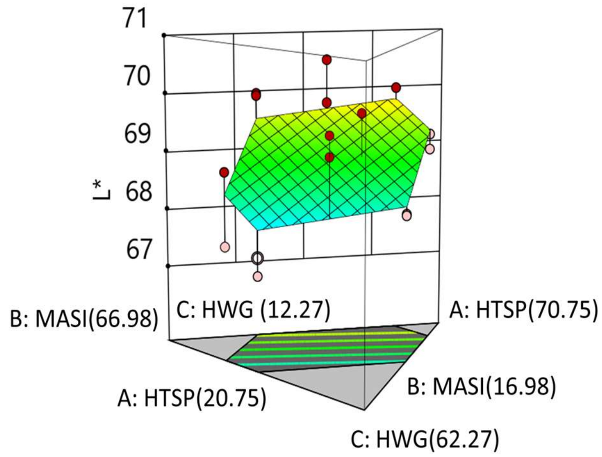

(b)

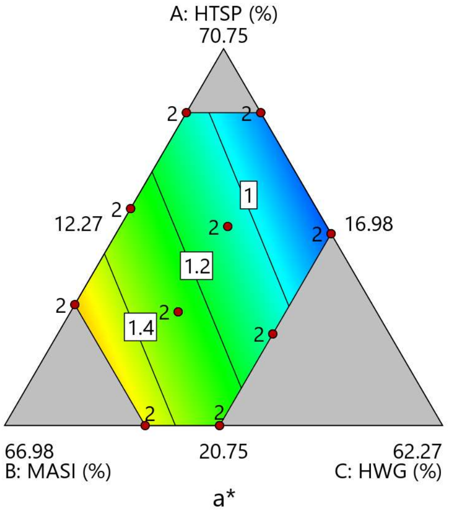

(c)

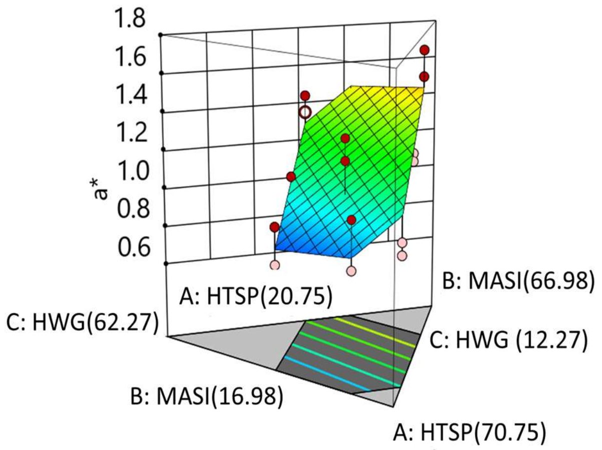

(d)

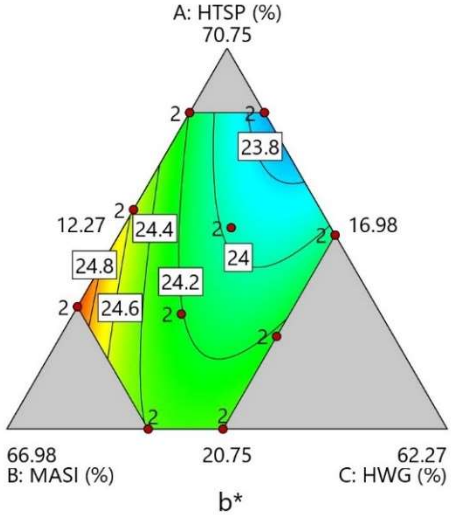

(e)

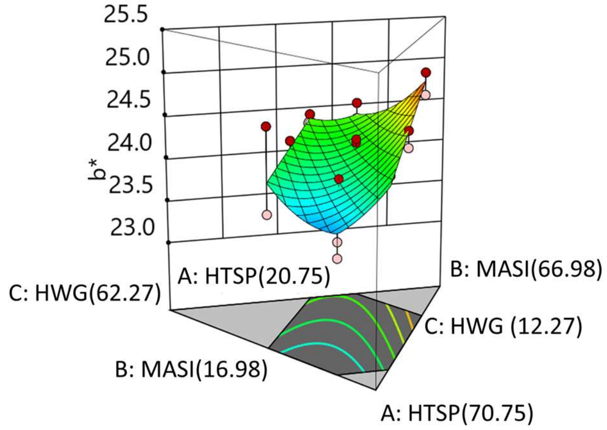

(f)

Figure S2. *Cont.*

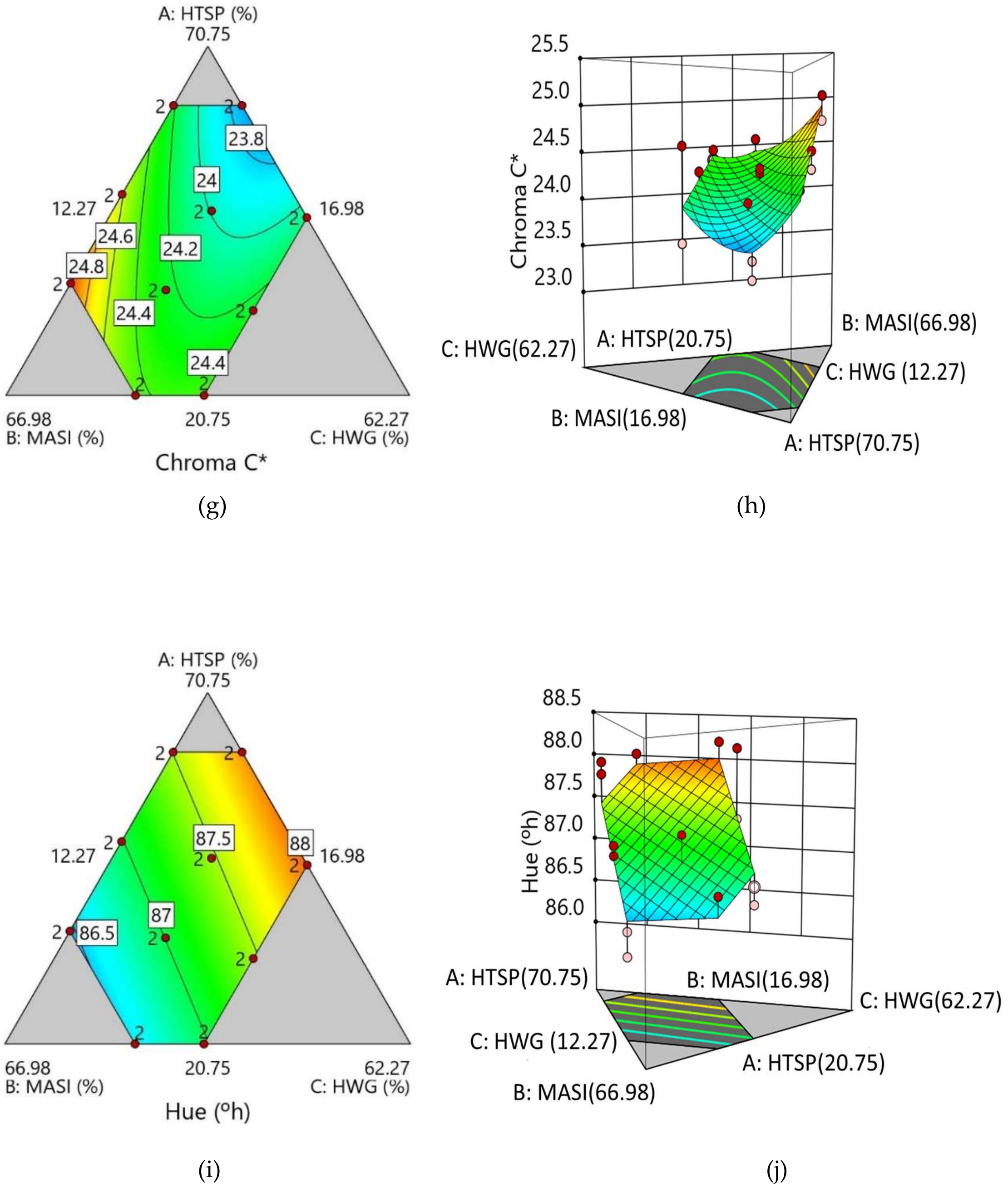

Figure S2: Two-dimensional contour surface (2-D, left) and three-dimensional surface (3-D, right) plots showing the effect of hydrated textured soy protein (HTSP, A), meat analogue based on extruded Sacha Inchi cake (MASI, B), and hydrated wheat gluten (HWG, C) on the color properties of the meat analogue nugget formulations, where: (a), L* (Lightness) 2-D; (b), L* (Lightness) 3-D; (c), a* (green–red) 2-D; (d), a* (green–red) 3-D; (e), b* (blue–yellow) 2-D; (f), b* (blue–yellow) 3-D; (g), C* (Chroma) 2-D; (h), C* (Chroma) 3-D; (i), °h (Hue angle) 2-D; and (j), °h (Hue angle) 3-D.

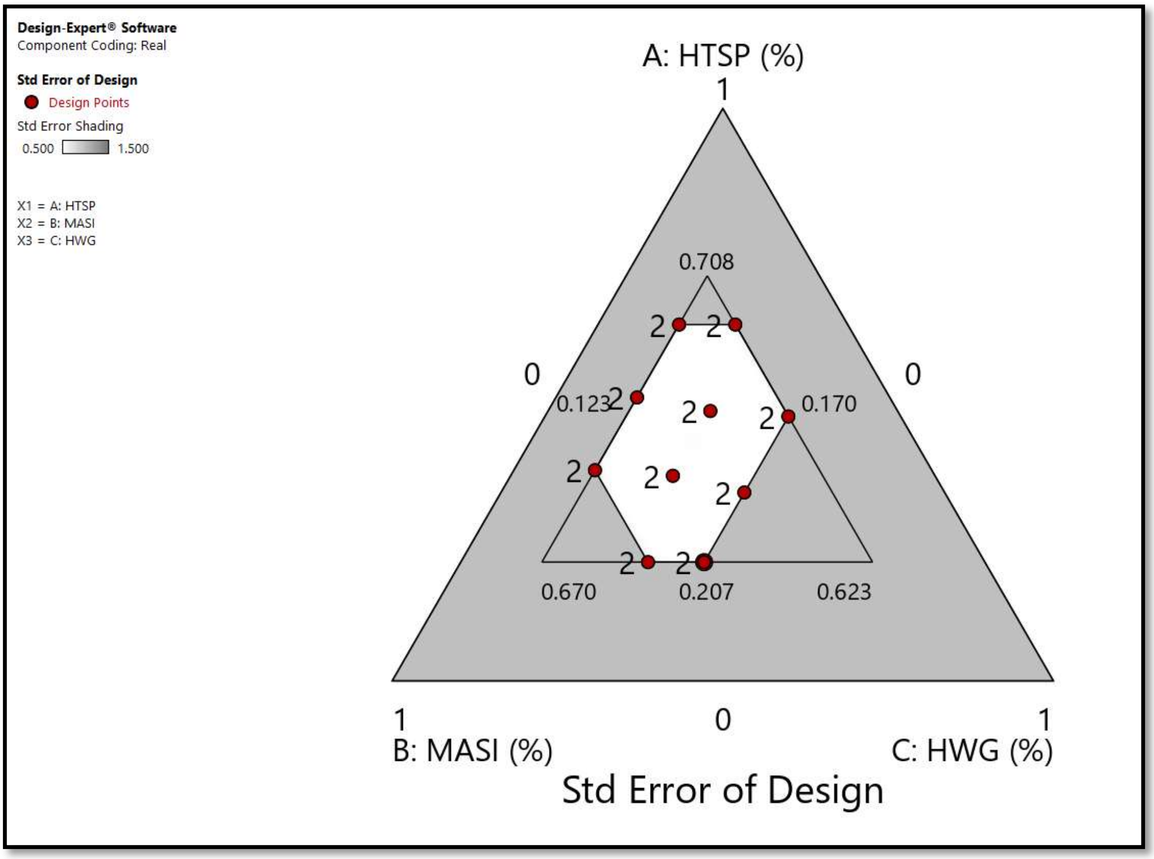

Figure S3: Experimental points within the experimental region of the constrained mixture design. HTSP: Hydrated textured soy protein; MASI: Meat analogue based on extruded Sacha Inchi cake; HWG: Hydrated wheat gluten. "2": The experiment was conducted in duplicate.

Table S1. Feasible formulations and desirability values.

Samples (solutions)	HTSP (%)	MASI (%)	HWG (%)	Springiness index	Hue (°h)	Desirability
1	20.75	46.25	33.00	0.350	86.79	0.710
2	20.75	50.93	28.32	0.327	86.57	0.699
3	34.76	28.44	36.81	0.362	87.54	0.506

HTSP: Hydrated textured soy protein; MASI: Meat analogue based on extruded Sacha Inchi cake; HWG: Hydrated wheat gluten. Desirability values, component proportions of feasible formulations, and predicted variable values.

Table S2. Variable Adjustment Limits for Optimization.

Name	Goal	Lower Limit	Upper Limit	Lower Weight	Upper Weight	Importance
A: HTSP	is in range	20.75	62.26	1	1	3
B: MASI	is in range	16.98	50.93	1	1	3
C: HWG	is in range	12.27	36.81	1	1	3
Hardness	none	4.74737	7.8335	1	1	3
Cohesiveness	none	0.128571	0.288889	1	1	3
Springiness Index	maximize	0.2	0.4	1	1	3
Chewiness Index	none	0.133571	0.628	1	1	3
Cooking loss	none	5.12413	9.76962	1	1	3
Water activity	none	0.9764	0.986167	1	1	3
Lightness L*	none	67.2763	70.5125	1	1	3
a*	none	0.742857	1.71833	1	1	3
b*	none	23.4586	25.0483	1	1	3
Chroma C*	none	23.4728	25.1077	1	1	3
Hue (°h)	minimize	86.0785	88.2103	1	1	3
Cooking yield	none	90.2304	94.8759	1	1	3
pH	none	6.6	6.74	1	1	3

HTSP: Hydrated textured soy protein; MASI: Meat analogue based on extruded Sacha Inchi cake; HWG: Hydrated wheat gluten. Adjustments for the optimization process of the optima formulation, maximizing the springiness index and minimizing the hue angle (°h). Lower and upper value limits are displayed.

Table S3. Cochran's Q test for each attribute in the nuggets.

Attributes	*p*	OF	FF	SF
Weak chicken odor	0.000	0.800 (b)	0.743 (b)	0.329 (a)
Intense chicken odor	0.000	0.057 (a)	0.057 (a)	0.357 (b)
Nugget aroma	0.000	0.100 (a)	0.186 (a)	0.600 (b)
Opaque mass color	0.000	0.543 (b)	0.414 (b)	0.029 (a)
Intense mass color	0.000	0.114 (a)	0.043 (a)	0.343 (b)
Thick breading	0.000	0.471 (b)	0.200 (a)	0.157 (a)
Thin breaded	0.000	0.100 (a)	0.343 (b)	0.400 (b)
Breading adhesion	0.044	0.286 (a)	0.157 (a)	0.157 (a)
Salty	0.001	0.043 (a)	0.086 (a)	0.200 (b)
Bitter	0.017	0.114 (b)	0.071 (ab)	0 (a)
Crunchy	0.000	0.814 (b)	0.414 (a)	0.457 (a)
Little crunchy	0.000	0.043 (a)	0.443 (b)	0.386 (b)
Hard	0.008	0.129 (b)	0.100 (ab)	0 (a)
Soft	0.000	0.586 (a)	0.471 (a)	0.800 (b)
Juicy	0.000	0.371 (b)	0.029 (a)	0.643 (c)
not very juicy	0.000	0.171 (a)	0.514 (b)	0.057 (a)
Greasy	0.000	0.100 (a)	0.043 (a)	0.343 (b)
Slightly greasy	0.001	0.471 (b)	0.471 (b)	0.214 (a)
Cardboard-like texture	0.000	0.157 (b)	0.314 (c)	0 (a)
Unpleasant flavor	0.013	0.114 (b)	0.057 (ab)	0 (a)
Tasty	0.000	0.086 (a)	0.114 (a)	0.614 (b)
Chicken flavor	0.000	0.043 (a)	0.200 (a)	0.657 (b)
Seasoned	0.000	0.157 (a)	0.086 (a)	0.543 (b)
Lightly seasoned	0.000	0.471 (b)	0.629 (b)	0.029 (a)
Cloying	0.292	0.043 (a)	0.043 (a)	0.100 (a)
Legume flavor	0.000	0.629 (c)	0.214 (b)	0.014 (a)
Meaty	0.001	0.129 (a)	0.186 (a)	0.343 (b)
Lumpy	0.001	0.171 (b)	0.214 (b)	0.014 (a)
Fibrous	0.004	0.200 (b)	0.200 (b)	0.029 (a)
Easy to chew	0.000	0.600 (a)	0.543 (a)	0.829 (b)

Types of nuggets: OF: Optimal Formulation; FF: commercial vegan nugget Flex Food; and SF: commercial chicken nugget San Fernando. Different letters in parentheses (a, b, and c) within the same row are significantly different ($p < 0.05$)

Table S4. Test of independence between rows and columns in CATA analysis.

Chi-cuadrado (Observed value)	582.366
Chi-cuadrado (Observed value)	74.468
GL	56
valor-p	< 0.0001
alfa	0.05

Table S5. Contingency values of the terms created from the CATA data.

Products\Dimensions	OF	FF	SF
Weak chicken odor	56	52	23
Intense chicken odor	4	4	25
Nugget aroma	7	13	42
Opaque mass color	38	29	2
Intense mass color	8	3	24
Thick breading	33	14	11
Thin breaded	7	24	28
Breading adhesion	20	11	11
Salty	3	6	14
Bitter	8	5	0
Crunchy	57	29	32
Little crunchy	3	31	27
Hard	9	7	0
Soft	41	33	56
Juicy	26	2	45
not very juicy	12	36	4
Greasy	7	3	24
Slightly greasy	33	33	15
Cardboard-like texture	11	22	0
Unpleasant flavor	8	4	0
Tasty	6	8	43
Chicken flavor	3	14	46
Seasoned	11	6	38
Lightly seasoned	33	44	2
Legume flavor	44	15	1
Meaty	9	13	24
Lumpy	12	15	1
Fibrous	14	14	2
Easy to chew	42	38	58

Types of nuggets: OF: Optimal Formulation; FF: commercial vegan nugget Flex Food; and SF: commercial chicken nugget San Fernando.
